# Molecular mechanisms of stress-induced reactivation in mumps virus condensates

**DOI:** 10.1016/j.cell.2023.03.015

**Published:** 2023-04-27

**Authors:** Xiaojie Zhang, Sindhuja Sridharan, Ievgeniia Zagoriy, Christina Eugster Oegema, Cyan Ching, Tim Pflaesterer, Herman K.H. Fung, Isabelle Becher, Ina Poser, Christoph W. Müller, Anthony A. Hyman, Mikhail M. Savitski, Julia Mahamid

**Affiliations:** 1 Structural and Computational Biology Unit, European Molecular Biology Laboratory (EMBL), Meyerhofstraße 1, 69117 Heidelberg, Germany; 2 Genome Biology Unit, European Molecular Biology Laboratory, Meyerhofstraße 1, 69117 Heidelberg, Germany; 3 Max Planck Institute of Molecular Cell Biology and Genetics, Pfotenhauerstraße 108, 01307 Dresden, Germany; 4 Cell Biology and Biophysics Unit, European Molecular Biology Laboratory, Meyerhofstraße 1, 69117 Heidelberg, Germany

**Keywords:** viral replication, persistent infection, biomolecular condensates, in-cell structural biology, whole-cell proteomics, phosphorylation, intrinsically disordered regions, IDR, nucleocapsid, cryo-focused ion beam, cryo-electron tomography

## Abstract

Negative-stranded RNA viruses can establish long-term persistent infection in the form of large intracellular inclusions in the human host and cause chronic diseases. Here, we uncover how cellular stress disrupts the metastable host-virus equilibrium in persistent infection and induces viral replication in a culture model of mumps virus. Using a combination of cell biology, whole-cell proteomics, and cryo-electron tomography, we show that persistent viral replication factories are dynamic condensates and identify the largely disordered viral phosphoprotein as a driver of their assembly. Upon stress, increased phosphorylation of the phosphoprotein at its interaction interface with the viral polymerase coincides with the formation of a stable replication complex. By obtaining atomic models for the authentic mumps virus nucleocapsid, we elucidate a concomitant conformational change that exposes the viral genome to its replication machinery. These events constitute a stress-mediated switch within viral condensates that provide an environment to support upregulation of viral replication.

## Highlights

• Cellular stress provokes activation of viral replication in an RNA virus model
• The viral replication factories are liquid-like condensates that coarsen under stress
• Stress-triggered IDR phosphorylation stabilizes the viral replication machinery
• In-cell nucleocapsid structures reveal changes in genomic RNA and IDR accessibility

## Graphical abstract

**Figure:**
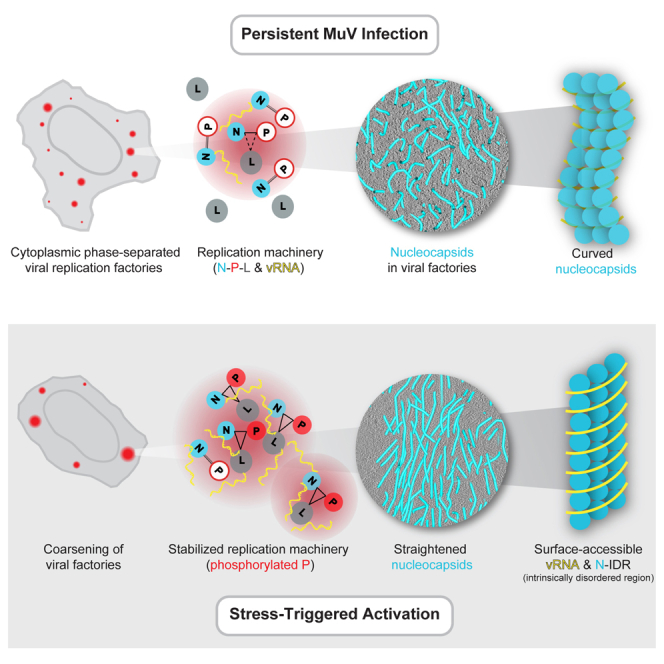

## Teaser

Cell biology, proteomics, and structural approaches uncover how stress to the host triggers replication and release of mumps virus during persistent infection via bimolecular condensation.

## Introduction

Long-term persistent viral infections are characterized by a metastable balance between viral replication and the host defense.^1^ Stress to the host has been implicated in tipping this balance in a few cases, including herpes simplex (HSV) and human immunodeficiency (HIV) viruses, and the emergence of pathology.^2^^,^^3^ However, the molecular mechanisms that underlie viral reactivation pertaining to stress remain elusive across the broad and divergent viruses. An accidental encounter of persistent viral infection in our cell cultures used for a stress response study allowed us to investigate the basis of stress-mediated reactivation in a member of the negative-stranded RNA virus family *Paramyxoviridae*, the mumps virus. Mumps is a highly contagious viral illness that was a common childhood disease before the introduction of vaccination.^4^^,^^5^ Mumps virus (MuV) is typically self-limiting. Namely, the majority of patients experience a full recovery and acquire lifelong immunity.^5^ However, chronic complications such as myositis,^6^ chronic arthritis,^7^ and encephalitis^8^ arise not due to an immediate effect of the primary infection (such as in parotitis^4^) but develop months or years thereafter or following vaccination in immunodeficient patients.^9^

The MuV RNA genome encodes for the nucleocapsid (N), viral/phospho- (V/P, co-transcriptional products), matrix (M), fusion (F), small hydrophobic (SH), hemagglutinin-neuraminidase (HN), and large (L) proteins. N self-assembles into a helical capsid that accommodates the RNA genome to form a nucleocapsid, which protects the viral genome from host nucleases and serves as the template for viral replication. Both genome transcription and replication are carried out by the viral RNA-dependent RNA polymerase L and require the phosphoprotein P that bridges between the nucleocapsid and the polymerase.^10^ The remaining MuV proteins are associated with nucleocapsid attachment to the inner side of the plasma membrane (HN), viral assembly (M), viral budding (F), and immunomodulation (V).

MuV transcription and replication occur in subcellular compartments termed viral factories (VFs), where the N-encapsidated viral genomes and replication machineries composed of P and L concentrate.^11^ The formation of VFs is a complex process that has independently evolved in a variety of non-related viruses.^12^ Biomolecular condensation mediated by liquid-liquid phase separation of viral proteins or host factors with intrinsically disordered regions (IDRs)^13^ and viral RNAs has been recently shown to be fundamental for the organization of VFs, regulation of viral replication, and promotion of viral assembly in measles virus (MeV),^14^ rabies virus,^15^ vesicular stomatitis virus,^16^ HIV-1,^17^^,^^18^^,^^19^ and SARS-CoV-2.^20^^,^^21^ MuV replication factories were reported to be membrane-less inclusions by conventional immuno-electron microscopy (EM) on sections of chemically fixed cells^11^ and typically contain curved tubule-like structures (the nucleocapsids) following viral inoculation into cultures to model acute infection. In contrast, biopsies from chronic myositis patient tissues show inclusions of straight nucleocapsids.^6^ Therefore, understanding the structural differences between these distinct nucleocapsid forms within intracellular VFs is important for gaining insights into their functional states and relevance to chronic diseases. However, such understanding has been critically limited by the low resolution that immuno-EM provides or the lack of biological context when obtaining high-resolution cryo-EM structures with recombinantly expressed and purified nucleocapsids.^22^

Here, we combine light microscopy imaging, whole-cell mass spectrometry (MS), cellular cryo-electron tomography (ET)^23^^,^^24^ following cryo-focused ion beam (FIB) thinning,^25^^,^^26^ and high-resolution structural analysis to provide a cross-scale characterization of a persistent viral infection model and demonstrate cellular, molecular, and structural changes that link upregulation of viral replication to exogenous stress to the host.

## Results

### Viral replication and release are accelerated by stress

**Figure 1:**
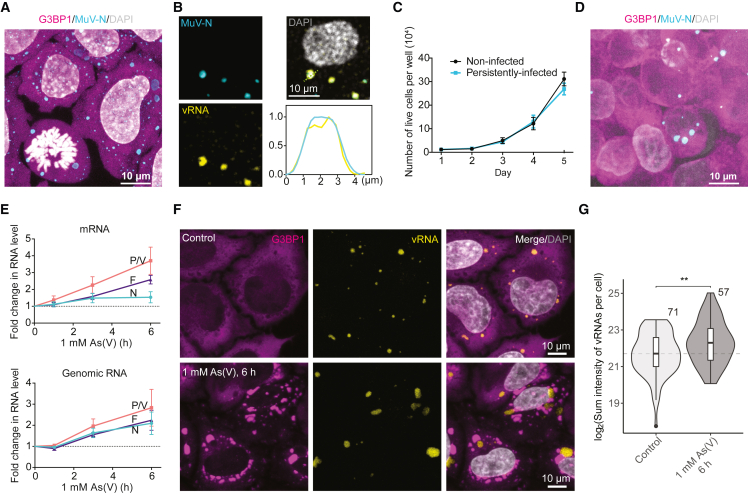
Persistent MuV infection model in HeLa exhibits viral reactivation upon stress (A) Maximum-intensity projection (MIP) of HeLa-MuV cells immuno-stained against the MuV-N protein (cyan). DNA stained with DAPI (gray). Note that persistent MuV infection does not trigger stress granule formation (mCherry-G3BP1 as a marker in magenta). (B) Immuno-RNA FISH of non-stressed HeLa-MuV cells. Central plane confocal images are shown. Intensity profile along the line in the merged panel shows colocalization of viral genomic RNA (yellow) and MuV-N (cyan). y axis is the intensity normalized to 0 and 1 for each channel. (C) Proliferation curves of HeLa-MuV cells in comparison with non-infected HeLa mCherry-G3BP1 cells. Data are shown as mean ± SD (n = 6). (D) MIP of naive HeLa mCherry-G3BP1 cells infected with MuV by transfer of medium from a persistently infected culture, imaged at 2 days post infection. (E) MuV transcription (mRNA) and replication (genomic RNA) levels determined by qPCR targeting different viral genes along the stress course. Dotted lines: starting levels. Data are mean ± SD (n = 3). (F and G) RNA FISH of HeLa-MuV cells at control and indicated stress condition and its quantification. Number of cells analyzed per condition is shown in the plot. Dashed line: median at the start of stress. Box center line: median; box bounds: the first and third quartiles; whiskers: values no further than 1.5 times the distances between the first and third quartiles; statistical significance evaluated using Wilcoxon test, ^∗∗^p < 0.01. See also Figure S1.

**Figure S1:**
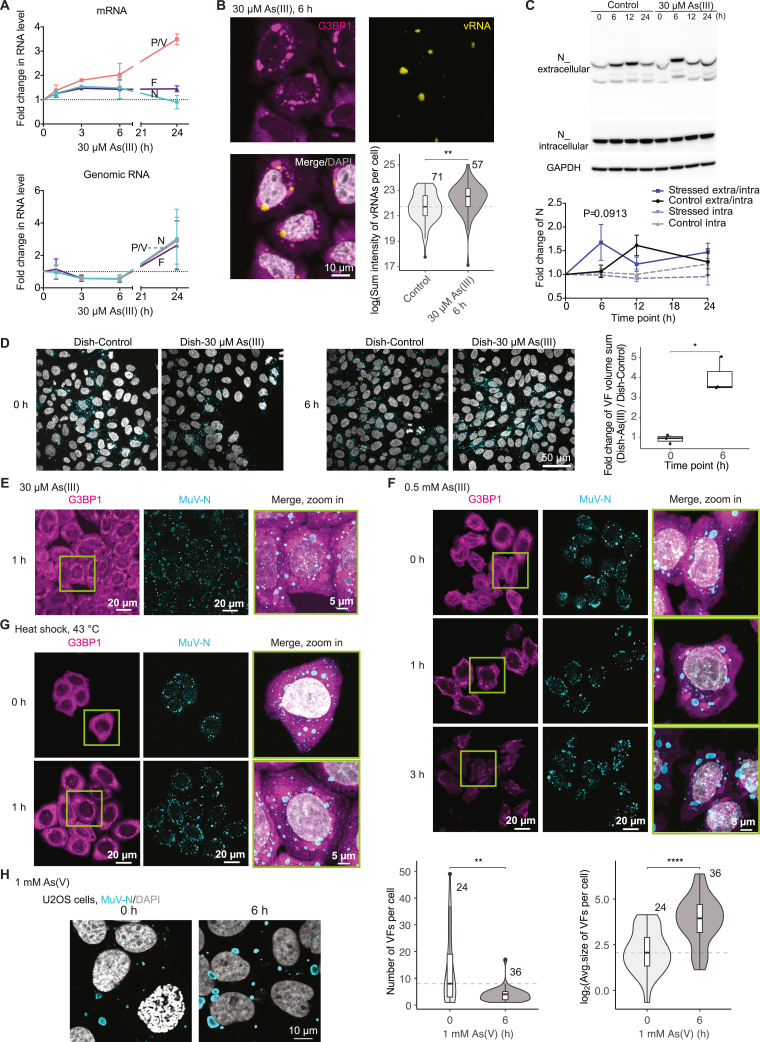
Effect of different stress conditions on viral reactivation in HeLa and U2OS cells, related to Figures 1 and 2 (A) MuV transcription (mRNA) and replication (genomic RNA) levels determined by qPCR targeting the N, P/V, and F genes during the time course of 30 μM As(III) mild stress. Dotted lines: starting levels. Data are mean ± SD (n = 3). (B) RNA FISH of HeLa-MuV cells at 6 h 30 μM As(III) stress (viral genomic RNA detected with single molecular FISH probes (vRNA; yellow)) and quantitative comparison to the non-stress control. Number of cells analyzed for each condition is shown on the plot. Box center line: median; box bounds: the first and third quartiles; whiskers: values no further than 1.5 times the distances between the first and third quartiles; dashed line: median at the start of stress. Statistical significance is evaluated using Wilcoxon test, ^∗∗^p < 0.01, ^∗∗∗^p < 0.001, and ^∗∗∗∗^p < 0.0001. (C) Representative western blot and quantification of MuV N protein levels in the cell culture medium as a proxy for virion release (N_extracellular) and inside cells (N_intracellular) during 24 h of 30 μM As(III) stress or of the non-stress control conditions collected at the same time points. Glyceraldehyde 3-phosphate dehydrogenase (GAPDH) was used as a loading control. Lower bands in N_extracellular are likely degraded forms of N in the medium.^111^ Ratio of MuV N_extracellular to N_intracellular (labeled as extra/intra for plots) and levels of N_intracellular (labeled as intra for plots) are normalized to levels at 0 h for each condition. Data are the mean ± SEM (n = 4). Note that levels in both experimental conditions oscillate over 24 h in agreement with Ruggieri et al.,^32^ with stress inducing a significant increase in released virions at 6 h. Statistical significance is evaluated between the stress and control using two-way ANOVA (Bonferroni’s multiple comparisons) and the label is the adjusted p value. (D) Images and quantification showing the effect of stress on infectious virus production. MIPs of naive HeLa mCherry-G3BP1 cells infected with MuV by transfer of medium from a persistently infected culture (at 0 and 6 h of 30 μM As(III) stress or non-stress control at the same time points), imaged at 2 days post infection. DAPI staining cell nuclei (gray) and anti-MuV N staining viral factories (VFs; cyan) are shown. The sum volume of VFs in the newly infected cells was normalized by the number of cells per image (approximated by the number of nuclei in the field of view), with the fold change of the stress condition over the non-stress control shown in the plot for the two time points (t test; n = 3). See also STAR Methods. (E–G) MIPs of VFs (detected by anti-MuV-N immunostaining) and stress granules (mCherry-G3BP1) in HeLa-MuV cells at 1 h of 30 μM As(III) stress (E; completing data presented in Figure 2B), during 3 h of 0.5 mM As(III) stress (F), and 1 h of heat shock (HS) at 43°C (G). DNA was stained with DAPI (gray). Quantification is provided in Figures 2D–2F. (H) MIPs of MuV factories in persistent MuV infection established in U2OS cells after 6 h of 1 mM As(V) stress in comparison with control and their quantification. Violin plots as defined above. Number of cells per condition is indicated. Statistical significance is evaluated using Wilcoxon tests.

Persistent infection of *Paramyxoviridae* can be established within weeks in various cell lines, including human amnion cells, lymphoid cells of B cell origin,^27^ and primary cells from human joint tissue.^7^ We established a culture model of persistent MuV infection in a HeLa cell line (HeLa-MuV) stably expressing the stress granule protein mCherry-G3BP1 (Ras GTPase-activating protein-binding protein 1) as a marker of cellular stress^28^ (Figure 1A; STAR Methods). Immunofluorescence using an antibody against the native viral N protein showed its localization in micron-sized cytoplasmic compartments that also contain the MuV genomic RNA (detected by RNA fluorescence *in situ* hybridization [FISH]; Figure 1B; Table S1), which we refer to as MuV replication factories. Our culture model recapitulated hallmarks of persistent benign infection^1^^,^^29^: cells did not exhibit stress as indicated by the absence of stress granules, and cell division was maintained at normal levels (Figures 1A and 1C), although released virions were capable of infecting naive cells, indicating basal levels of viral replication (Figure 1D). However, when cells were challenged with established conditions of oxidative stress,^28^^,^^30^ viral replication increased by 2- to 4-fold (Figures 1E and S1A). Specifically, transcription levels probed by quantitative PCR (qPCR; Table S1) of P/V and F genes steadily increased over 6 h under severe stress (1 mM arsenate; As(V)) and that of N increased to a plateau after 3 h (Figure 1E) in agreement with a previous report showing that ectopic expression of MeV N limited its own viral replication.^31^ Viral replication, inferred from the genomic RNA level, was similarly upregulated (Figure 1E). Prolonged mild oxidative stress (30 μM arsenite; As(III)) also increased viral transcription and replication, albeit at a lower rate or magnitude (Figure S1A). Thus, global increase in viral activity correlated with the severity of stress. Quantification of viral genomic RNA levels inside the VFs by RNA FISH also demonstrated an increase in stress (1.6-fold at 6 h of mild or acute stress; Figures 1F, 1G, and S1B). We additionally measured N protein levels in the media as a proxy for virion release. Although extracellular N protein levels oscillated over 24 h, as has been reported for the hepatitis C virus,^32^ stress accelerated viral budding by 1.7-fold and led to 3.5-fold higher infectious virus production at 6 h of mild stress compared with the non-stressed condition (Figures S1C and S1D). These results substantiate previous reports on the effect of stress on viruses from distant families^2^^,^^3^ and the closely related MeV,^33^ confirming that cellular stress increases viral transcription, replication, and infectious virion release in a persistent MuV infection culture model.

### Persistent MuV factories are dynamic condensates that coarsen under stress

**Figure 2:**
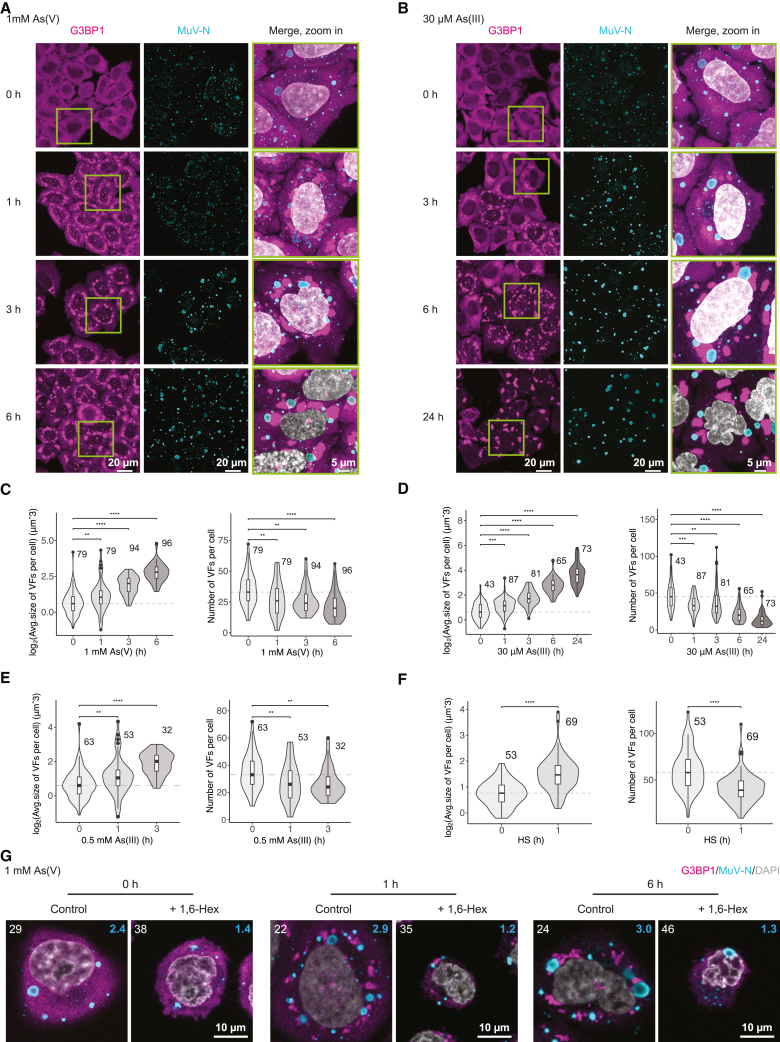
Persistent MuV replication factories coarsen under different stress conditions (A and B) MIPs of MuV factories (VFs; detected by anti-MuV-N immunostaining) and stress granules (mCherry-G3BP1) in HeLa-MuV cells during the time course of acute and mild stress. (C–F) Quantification of data in (A) and (B) and two additional stress conditions for VF size and number per cell. Number of cells per condition is indicated. HS, heat shock. Violine plot details as above. Statistical significance evaluated using Wilcoxon test, ^∗∗^p < 0.01, ^∗∗∗^p < 0.001, and ^∗∗∗∗^p < 0.0001. (G) Effect of 15 min 1,6-hexanediol (1,6-Hex) treatment on authentic MuV factories over the time course of stress. HeLa-MuV cells were stressed to the indicated time points and then incubated with 1,6-Hex or water as control. Central plane images are shown. Upper left corresponds to the number of cells per condition; upper right is the average number of VFs larger than 2 μm in diameter per cell at each condition. See also Figure S1.

To investigate the cellular mechanisms of the stress-induced MuV activation, we monitored changes to the MuV factories, the location of viral replication, using immunofluorescence labeling of the native viral N protein along the course of cellular stress (Figures 2A, 2B, and S1E–S1G). Both oxidative stress and heat shock elicited stress granules as a part of the cellular response^28^^,^^30^ and resulted in an increase in VFs size, concurrently with a decrease in their numbers (quantified in Figures 2C–2F). We validated that the effect was not unique to HeLa cells by reproducing the MuV persistent infection and stress-induced changes in VFs in U2OS cells (Figure S1H). The increase in VF sizes and decrease in their numbers is reminiscent of the coarsening behavior described for liquid-like biomolecular condensates, whereby larger droplets grow at the expense of smaller ones by fusion or Ostwald ripening (dissolution and growth).^34^^,^^35^^,^^36^ Indeed, treatment with hexanediol previously shown to disrupt weak multivalent interactions in cellular condensates^20^ led to dissolution of the liquid-like stress granules^37^ in our MuV model and also dissolved the authentic VFs to a large extent (40–60% in the number of large VFs; Figure 2G). These data suggested that MuV factories may be biomolecular condensates.

**Figure 3:**
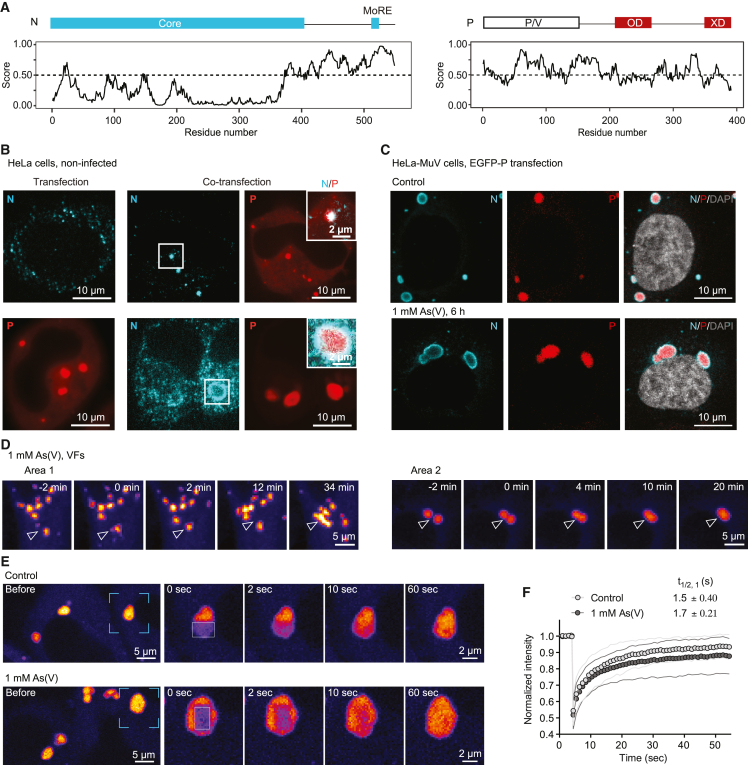
The MuV phosphoprotein drives condensation of dynamic replication factories (A) Domain architecture of viral N and P proteins. Intrinsically disordered regions (IDRs) were predicted with IUPred2A (residues with scores higher than 0.5). N-Core, structural core of N; N-MoRE, region interacting with P-XD domain; P/V region, shared between P and V proteins; P-OD, P oligomerization domain. (B) Transfection of MuV N (detected by immunostaining) or EGFP-P, or their co-transfection into naive HeLa cells. (C) Immunostaining of the authentic MuV-N in HeLa-MuV cells transfected with EGFP-P under non-stress control and indicated stress condition. Central plane images are shown. (D) MIPs of two representative areas showing fusion events of VFs of different sizes in HeLa-MuV cells transfected with EGFP-P during stress treatment. Images captured within 3 h of stress. The time of condensates touching considered as time 0. (E and F) Partial photobleaching of VFs in HeLa-MuV cells transfected with EGFP-P at the non-stress control or within 6 h of stress. Framed areas: individual VFs and zoom-in views shown on the right; boxed areas: bleach regions of EGFP-P within VFs. Quantification shows the normalized mean fluorescence recovery curve (connected circles) and SD (thin lines), n = 12 for control and n = 15 for 1 mM As(V) stress. Half-life time is shown as mean ± SD. See also Figure S2 and STAR Methods.

**Figure S2:**
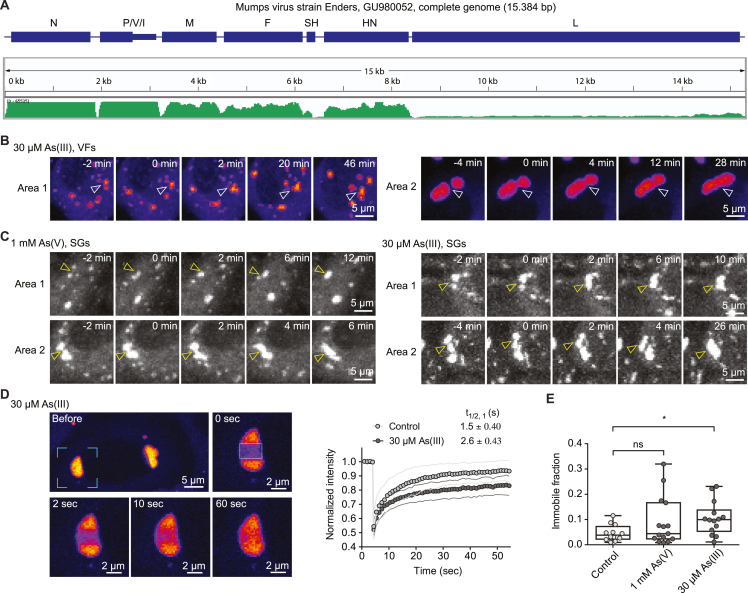
Expression levels of MuV genes and dynamic properties of VFs in HeLa-MuV cells, related to Figure 3 (A) Transcriptome sequencing of HeLa-MuV cells after ∼1 h of 0.5 mM As(III) stress. Transcripts were mapped to the MuV genome (top, bule) to reveal its transcription profile (bottom, green). The unit of the coverage tracks is number of reads per genomic position, scaled at 0–65535. P/V/I are co-transcriptional products; little is known about the functions of V/I proteins and they are not discussed in this study. (B) Fusion events of MuV factories (VFs) of different sizes in HeLa-MuV cells transfected with EGFP-P during mild stress acquired by time-lapse imaging. Images were captured within 3 h of 30 μM As(III) stress treatment, similar to representation shown in Figure 3D. (C) Fusion events of stress granules (SGs) of different sizes in HeLa-MuV cells within the same experiments shown in Figures 3D and S2B. (D) Fluorescence images and quantification of partial photobleaching of VFs in HeLa-MuV cells transfected with EGFP-P. Bleaching was performed at up to 6 h after the start of 30 μM As(III) mild stress, similar to representation shown in Figure 3E. Quantification is similarly shown as in Figure 3F, n = 14 for 30 μM As(III) stress. (E) Immobile fraction of EGFP-P within the bleached areas, obtained with data shown in Figures 3E, 3F, and S2D. Each dot corresponds to one recovery curve fitted. Statistical significance is evaluated using Student’s t tests.

Biomolecular condensation is often driven by multivalent interactions, commonly mediated by IDRs.^13^^,^^14^ In fact, the MuV N and P genes, which we found to be the most highly transcribed among the viral genes in our persistent infection model (Figure S2A), encode sequences indicative of disordered regions (Figure 3A).^38^^,^^39^^,^^40^ To test whether N or P proteins are sufficient for condensation, we transfected untagged N and enhanced green fluorescent protein (EGFP)-tagged P genes into naive HeLa cells. Although N-only transfection resulted in small puncta scattered throughout the cytoplasm (Figure 3B, upper left), P transfection resulted in the formation of spherical cytoplasmic condensates (Figure 3B, bottom left). Co-transfection of P with N led to co-condensation of both proteins (Figure 3B, right). Thus, the largely disordered P protein can drive condensation of MuV replication factories and promote sequestration of nucleocapsids. Next, we sought to monitor the dynamic properties of the condensed authentic VFs in the functional context of all viral proteins and RNA. Due to the difficulty in engineering negative-stranded RNA viruses^41^ and caveats in establishing functional fluorescently tagged viral proteins, we opted to transfect a reporter viral gene fused to a fluorescent tag into the HeLa-MuV cells. In our trial to tag the main VF components (N, P, or L) with fluorescent proteins, only that of the P resulted in successful expression (Figure 3B), suggesting interference in folding of N and L by the presence of a fluorescent protein tag. The transfected EGFP-tagged P protein selectively partitioned into the authentic MuV factories at both control and stress conditions (Figure 3C). This allowed us to observe fusion of MuV factories under both acute and mild stress (Figures 3D and S2B; comparison to stress granules shown in Figure S2C), albeit with dynamics on the scale of tens of minutes indicative of high viscosity. Fast internal rearrangement was nevertheless indicated by a high extent of fluorescence recovery after photobleaching (FRAP) within 1–3 s at both control and stress conditions (Figures 3E, 3F, S2D, and S2E). Taken together, these results show that MuV factories during persistent infection are dynamic condensates that coarsen under stress in concomitance with viral reactivation.

### Stress-induced alterations in protein interactions stabilize the MuV replication machinery

**Figure S3:**
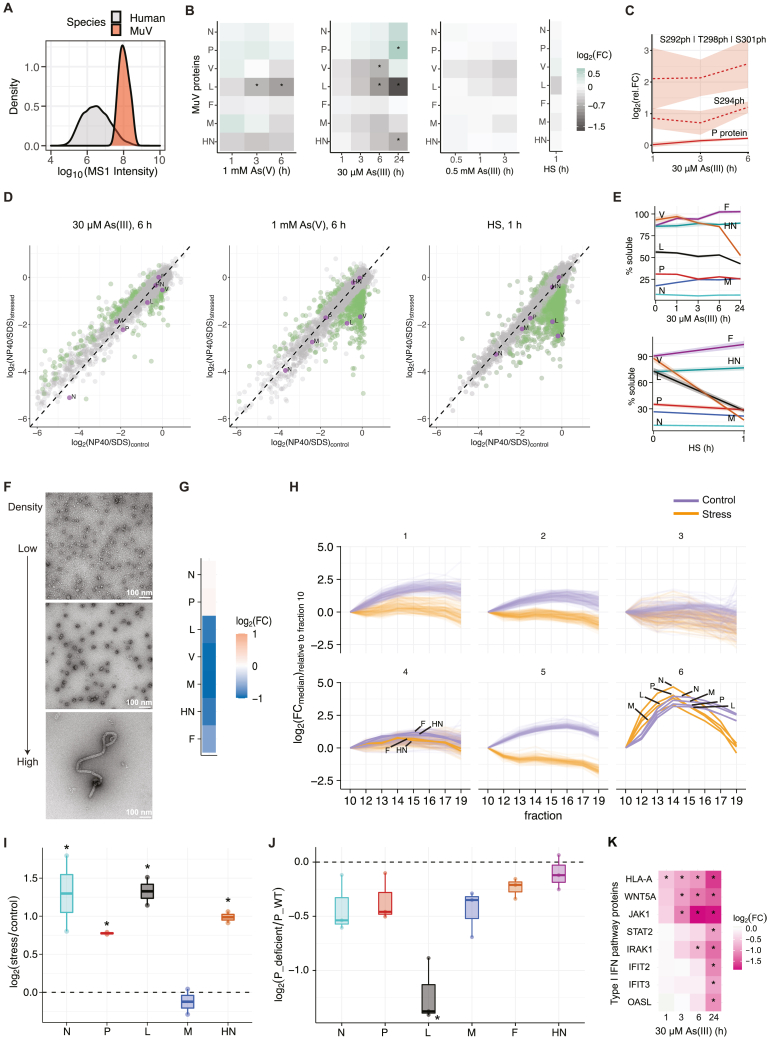
Proteomics of HeLa-MuV, related to Figure 4 (A) Density plot representing the distribution of MS1 intensity of the MuV and human proteomes in non-stressed HeLa-MuV cells. (B) Heatmap representation of the relative fold change (FC) of all MuV proteins inside persistently infected cells at indicated time points of 1 mM As(V), 0.5 mM As(III), or 30 μM As(III) stress, or heat shock (HS) stress at 43°C, in comparison with the non-stress control (n = 3). Asterisks indicate proteins with |log_2_(fold change)| > 0.5 and adjusted p value < 0.01 (Benjamini-Hochberg method). (C) Relative fold change of P along 30 μM As(III) stress time course relative to control: unmodified protein (solid line) and phosphopeptides (dashed lines). Lines and shaded areas are mean and SEM (n = 3). See also Figure 4A. (D) Scatter plots representing the solubility of viral and host proteins in non-stress control cells (x axis) and under the indicated stress condition (y axis). Solubility is defined by NP40/SDS ratio, indicating the proportion of soluble protein in the lysate pelleting assay. Purple dots: viral proteins; green dots: host factors that exhibited significant change in solubility; gray dots: host factors that did not exhibit significant change in solubility. Proteins with |log_2_(fold change)| > 0.5 and adjusted p value (Benjamini-Hochberg method) < 0.1 were considered significantly changed. Data are mean values (n = 2). (E) Mean solubility profiles (line) and SEM (shaded area; n = 2) of all MuV proteins along 30 μM As(III) mild stress time course, or after acute heat-shock stress, related to Figure 4B. (F) Representative negative-staining transmission electron microscopy (TEM) images of fractions from sucrose gradient fractionation experiments for isolating nucleocapsids from the 13,000 g pellet of the HeLa-MuV cell after ∼1 h of 0.5 mM As(III) stress to the cells. (G) Heatmap representation of relative fold change (in log_2_ scale) of all MuV proteins in 13,000 g pellet (input of sucrose gradient fractionation) after 6 h of 1 mM As(V) stress in comparison with the non-stress control, used for normalization of data in (H). Median values are shown (n = 3). (H) Hierarchical clustering of sucrose gradient fractionation profiles (median relative fold change compared with fraction 10, n = 3) of all proteins in non-stress control and 6 h of 1 mM As(V) stress. Thick lines: MuV proteins; thin lines: HeLa proteins. See also Figures 4D and 4E. (I) Fold change of viral proteins in anti-EGFP-P pull-down from cells transfected with P_WT at 6 h of 1 mM As(V) stress compared with non-stress control, quantified by MS (n = 2). Statistical significance of change evaluated as in (D). (J) Comparison of the amount of viral proteins in anti-EGFP-P pull-down between P_deficient and P_WT at non-stress condition, quantified by MS (n = 3). Statistical significance of change evaluated as in (D). (K) Heatmap representation of relative fold change of proteins from the type I interferon signaling pathway (HeLa-MuV cells) shows significant down-regulation along the stress time course compared with the non-stress control (n = 3). Asterisks indicate proteins with |log_2_(fold change)| > 0.5 and adjusted p value < 0.01 (Benjamini-Hochberg method).

**Figure 4:**
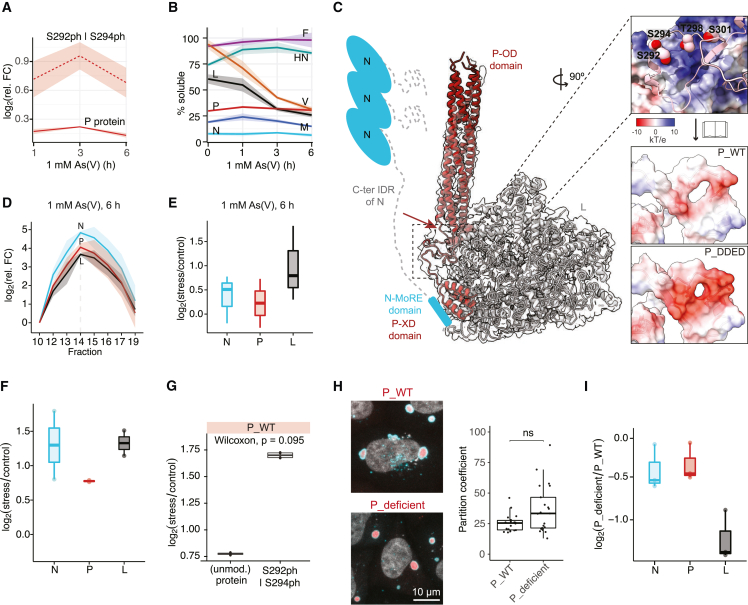
Stress alters protein interaction networks within viral factories (A) Mean relative abundance of P protein (solid line) and its phosphopeptides (dashed line) in HeLa-MuV cells along the stress course in comparison with 0 h control in log_2_ scale. Shaded areas represent SEM (n = 3). (B) Mean solubility profiles (line) and SEM (shaded area; n = 2) of all MuV proteins in HeLa-MuV cells along the stress course. (C) Structural model of the P-L interface generated with AlphaFold2 (AF2). Left: AF2 predicted model of PIV5 P-L fitted into the cryo-EM map (EMD: 21095) positions the P-IDR at the previously unassigned density (framed; the region corresponding to the MuV-P phosphopeptide in A is indicated with red arrow). Schematic model of N is shown to indicate the context of P-L complex, with a short helix (MoRE) at the C-terminal IDR of N predicted to interact with the P-XD. Right: The zoom-in view of the MuV P-L interface (top) is shown with L in surface charge representation and P in cartoon. Surface charge comparison of the same interface on P-WT versus a mimic of phosphorylated P (S|T mutated to D|E; P_DDED) is shown below. (D) Mean relative abundance of N, P, and L proteins (line) in sucrose gradient fractions, with respect to fraction 10 at stress condition. Apex of co-eluting proteins N, P, and L is observed at fraction 14. Shaded areas: SEM (n = 3). (E) Relative abundance of N, P, and L at the apex of stress condition compared to non-stress control (n = 3). (F) Fold change of N, P, and L in anti-EGFP-P pull-down from cells transfected with P_WT at 6 h of 1 mM As(V) stress compared to non-stress control, quantified by MS (n = 2). (G) Fold change of P phosphopeptide enriched in anti-EGFP-P pull-down in (F). (H) Comparison of partitioning of EGFP-tagged P_WT and P_deficient transfected into HeLa-MuV cells. Cyan: VFs immunostained against MuV-N; gray: DNA DAPI staining; red: EGFP-P. MIP images are shown. Each dot in the plot represents one cell. Statistical significance is evaluated using Wilcoxon test. ns, not significant. (I) Comparison of the amount of N, P, and L proteins in anti-EGFP-P pull-down between P_deficient and P_WT at non-stress condition, quantified by MS (n = 3). See also Figure S3.

To elucidate the molecular changes that take place within the dynamic MuV factories upon stress, and which may support the measured increase in MuV replication, we performed whole-cell measurements of proteins’ abundance and solubility (i.e., their propensity to undergo phase transition) using quantitative MS-based approaches.^42^ First, whole-cell abundance proteomics showed that non-stressed cells expressed high levels of MuV proteins (Figure S3A), which surprisingly exhibited only modest changes in intracellular levels under all examined stress conditions (Figure S3B; STAR Methods). However, despite overall stable level of P protein—the main driver of MuV VF condensation—levels of P phosphopeptides spanning residues 292–328 within its IDR were significantly elevated during stress (S292|S294|T298|S301, 2-fold in Figure 4A and up to 6-fold in Figure S3C, depending on the stress; STAR Methods). In parallel, we used a lysate pelleting assay coupled with quantitative MS to assess viral protein solubility at the whole-cell level.^42^^,^^43^ The solubility profiling revealed that significant proportions of the viral proteins N (∼90%), M (∼80%), and P (∼70%) resided in the insoluble fraction in unstressed cells (Figures 4B, S3D, and S3E; STAR Methods). This is consistent with the documented propensity of N and M to polymerize into structured assemblies^44^ and of P to assemble into condensates as shown above (Figure 3B). All other viral proteins were predominantly soluble in unstressed cells, suggesting they may only interact transiently with the core VF proteins N and P.^45^ L, however, exhibited a significant decrease in solubility especially under severe stress (Figures 4B, S3D, and S3E). Such a change in solubility suggested increased partitioning of L into the viral condensates and thereby could explain the increase in viral transcription and replication under cellular stress. Seeing as the decrease in L solubility correlated with an increase in P phosphorylation, we hypothesized that the two events may be related to the molecular interactions between the two viral proteins.

To investigate this possibility, we first examined the reported structure of the P-L complex from a closely related parainfluenza virus 5 (PIV5) ^46^ and predicted a model for the MuV complex using AlphaFold2.^47^^,^^48^ Both models showed that amino acid residues of P (S292, S294, T298, and S301), which gained phosphorylation under cellular stress in our measurements, were located within a disordered sequence at its interaction interface with L (Figure 4C, left). P and L presented complementary charges at this interface (Figure 4C, top right). Hence, we hypothesized that further increasing the negative charge of this IDR of P through phosphorylation may stabilize the P-L complex (Figure 4C, right). To experimentally verify this, we performed sucrose gradient fractionation of the HeLa-MuV cell lysate coupled with quantitative MS and observed nearly a 2-fold enrichment of L in the N-P-L complex under stress (Figures 4D, 4E, and S3F–S3H; STAR Methods). We further substantiated this with an independent experimental strategy by an affinity purification-MS assay using EGFP as bait in HeLa-MuV cells transiently transfected with EGFP-P (Figure 3C). We found a ∼2.5-fold increase for L co-purification after stress and overall stabilization of the N-P-L complex in line with the sucrose gradient experiments (Figures 4F and S3I). More interestingly, we re-searched our pull-down-MS data including phosphorylation as a potential modification and found a higher amount of P-phosphopeptide spanning residues 282–328 (with phosphorylation on S292|S294) bound to EGFP-P compared with the unphosphorylated P-protein after stress (Figure 4G; STAR Methods). This shows that phosphorylated P is found in higher abundance within the N-P-L complex under stress. The two independent experiments support a model wherein stress leads to the stabilization of interactions between N, P, and L, likely involving phosphorylation of P, and suggest that the negative charges on P at this interface are important for its interaction with L.

To establish a direct link between charge complementarity at the P-L interface, the contribution of P phosphorylation at this location, and stabilization of the N-P-L complex under stress, we generated a phospho-deficient version of P by substituting S292, S294, T298, and S301 with alanine (P_deficient) and expressed it as an EGFP-fusion protein in HeLa-MuV cells. Confocal imaging and quantification showed comparable partitioning of P_deficient into the authentic VFs to the wild type (P_WT; Figure 4H). Affinity purification-MS assay (using EGFP as a bait) showed a significant reduction in the amount of L bound to P_deficient compared with the P_WT (∼2.3-fold lower; p value = 0.00026; STAR Methods), whereas interactions with all other viral proteins did not change significantly (Figures 4I and S3J). Therefore, loss of charge complementarity and abolishing phosphorylation in this region of P significantly impact its association with L. Indeed, structural modeling of the P-L interface with a mimic of the phosphorylated P (mutating S to D and T to E in the S292–S301 region) moves this segment toward L with its surface charge indicating a stronger potential to support their interaction upon phosphorylation (Figure 4C, bottom inset). Combining our experimental and modeling evidence, we suggest that phosphorylation of P by host factors is a potential molecular mechanism for stress-induced stabilization of L with the MuV nucleocapsids that harbor the viral RNA, thereby providing conditions to support upregulation of viral transcription and replication.

In addition to changes that directly affect the viral replication machinery, quantitative MS of the host proteome revealed concomitant changes in the host immune response. Although attenuation of the host antiviral defense by degradation of the signal transducer and activator of transcription 1 (STAT1) has been documented in persistent infection,^49^^,^^50^ we additionally found a significant downregulation of type I interferon (IFN) signaling pathway proteins over 24 h of stress treatment, including the mediators Janus kinase 1 (JAK1) and STAT2 from the start of stress (Figure S3K; STAR Methods). Stress-mediated viral reactivation therefore appears to be supported by downregulation of the host innate defense, by mechanisms that remain to be elucidated in detail, in addition to the changes in the interaction networks within the viral condensates.

### Stress induces a conformational switch in the MuV nucleocapsids

**Figure S4:**
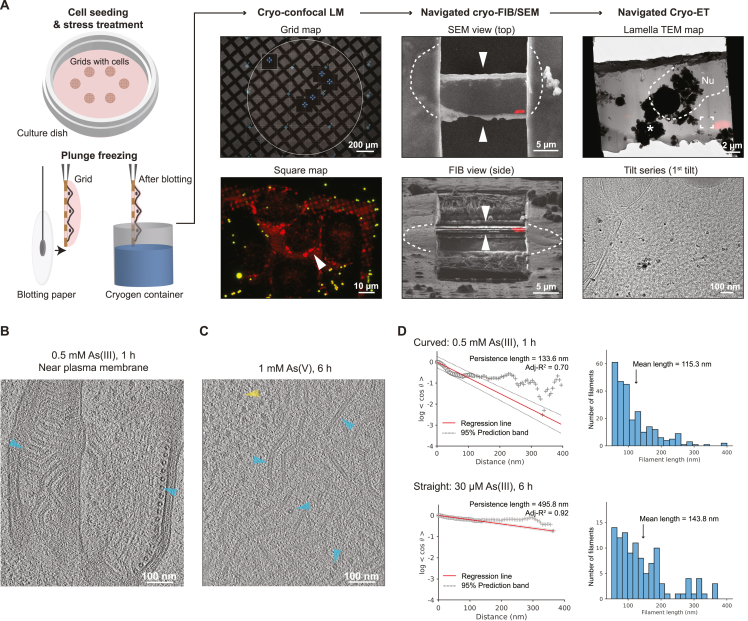
Workflow of *in situ* cryo-correlative electron tomography and tomogram analysis, related to Figure 5 (A) 3D cryo-correlative light and electron microscopy (CLEM) workflow targeting unlabeled MuV factories in vicinity of stress granules (SGs). MuV factories were frequently seen in proximity of SGs identified by mCherry-G3BP1 (see also Figures 2A and 2B). HeLa-MuV cells were seeded and cultured on TEM grids in a culture dish. At different time points of stress, grids with cells were plunge-frozen, followed by imaging with a cryo-confocal light microscope (LM). Grid squares with cells showing SG signals (red; indicated with white arrowhead) and optimal fluorescent beads (yellow) distribution in grid map overviews were chosen for imaging in confocal mode. Lamellae were then prepared by cryo-FIB (focused ion beam) milling at SG locations, using the fluorescent beads as fiducials for 3D correlation between LM and scanning electron microscopy (SEM)/FIB images. Correlating SEM/FIB images with 3D LM signals after lamella preparation confirmed SG location preservation (red signal) in lamella. Overlaying the SEM image with transformed fluorescent signal onto the cryo-TEM image of the lamella allowed identification of SG location and informed tilt series acquisition (indicated by frame) at nearby regions where filamentous structures were seen and later identified to be MuV nucleocapsids. Nu: nucleus. Asterisk: ice contaminants introduced during transfers between the cryo-microscopes. (B) A 6.74 nm-thick tomographic slice of nucleocapsids aligned near the plasma membrane. Cyan arrowheads: nucleocapsids. (C) Representative tomographic slice showing MuV factories at 1 mM As(V) stress. Cyan arrowheads: nucleocapsids (both top and side views, two of each); yellow arrowheads: ribosomes. Slice thickness: 6.52 nm. See also Figure 5C. (D) Examples of persistence length measurement (left) and histogram of nucleocapsid length (right) derived from tracing of all nucleocapsids in two tomograms representing curved and straight nucleocapsids, obtained at indicated stress conditions. 3D coordinates along all traced nucleocapsids within one tomogram were used to calculate the “tangent-correlation length” (apparent persistence length in the filament ensemble), which is inversely related to the curvature of filament (see also STAR Methods). Mean length of filaments per tomogram was determined to define the length range used for fitting. See also Figure 5D for quantification of nucleocapsid persistence length measurements in all tomograms generated in this study.

**Figure 5:**
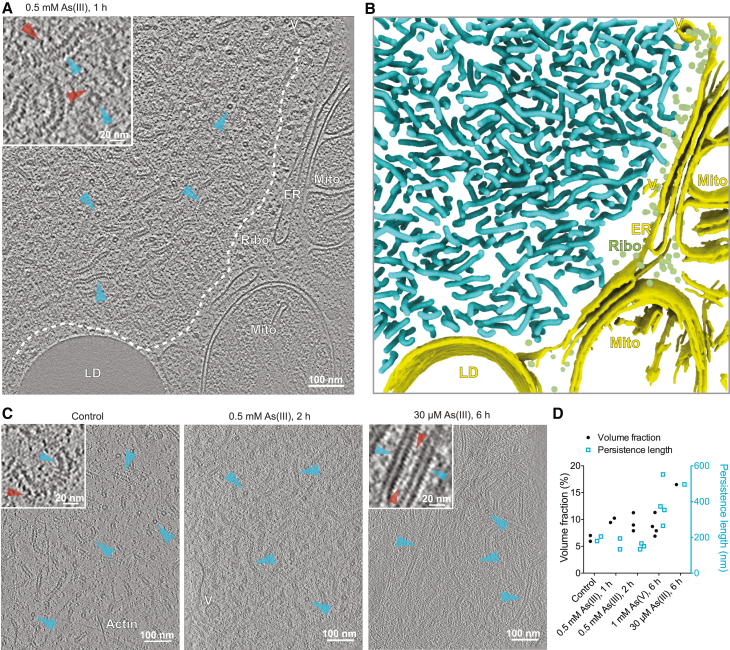
MuV viral factories and nucleocapsids undergo structural changes during stress (A and B) 6.74-nm-thick tomographic slice of a VF and the corresponding 3D segmentation. Exemplary nucleocapsids are indicated with cyan arrowheads (top and side views, two of each) in (A) and colored the same in (B). Inset: enlarged view. Red arrowheads indicate flexible densities extending from nucleocapsids. LD, lipid droplet; Mito, mitochondrion; ER, endoplasmic reticulum; Ribo, ribosome; V, vesicle. (C) Representative tomographic slices (thickness left to right: 6.74, 6.52, and 6.52 nm) of nucleocapsids (cyan arrowheads) and associated flexible densities (red arrowheads in insets) in VFs at indicated control and stress conditions. (D) Volume fractions and apparent persistence lengths of nucleocapsids in tomograms at indicated control and stress conditions. See also Figure S4 and Videos S1, S2, and S3.

To structurally assess the consequences of the stress-mediated cellular and molecular changes, we performed cryo-ET on the intracellular authentic VFs. MuV factories frequently localized in proximity to stress granules (Figures 2A and 2B). We thus exploited the mCherry-G3BP1 fluorescence to perform 3D targeted cryo-FIB thinning and to locate unlabeled VFs for cryo-ET (Figure S4A). We observed curved hollow filaments resembling low-resolution maps of MuV nucleocapsids^10^^,^^51^ within VFs (Figures 5A and 5B) and near the plasma membrane (Figure S4B). 3D tracing of the nucleocapsids allowed the quantitative analysis of changes in molecular crowding along the stress course (Figure 5C; Videos S1, S2, and S3) and showed that nucleocapsid volume fractions in the VFs tripled from the control to 6 h of mild stress (30 μM As(III); Figures 5C, 5D, and S4C). In addition, 2.5–3 nm thick flexible densities extending from the nucleocapsids were abundant under stress conditions (Figures 5A and 5C), potentially marking interaction partners of N. Strikingly, prolonged stress led to a morphological transition from curved to straight nucleocapsids (Figures 5C, 5D, and S4D) with the latter reminiscent of aberrant MuV inclusions in myositis patient biopsies.^6^

Video S1 Cryo-ET of a viral factory in a HeLa-MuV cell and 3D tracing of nucleocapsids at non-stress condition, related to Figure 5
Data were acquired with a Volta phase plate and defocus. Scale bar: 100 nm. Tomographic slice thickness: 6.74 nm.

Video S2 Cryo-ET of a viral factory in a HeLa-MuV cell and 3D tracing of nucleocapsids at 1 h of 0.5 mM As(III) stress, related to Figure 5
Data were acquired with a Volta phase plate and defocus. Scale bar: 100 nm. Tomographic slice thickness: 6.74 nm.

Video S3 Cryo-ET of a viral factory in a HeLa-MuV cell and 3D tracing of nucleocapsids at 6 h of 30 μM As(III) stress, related to Figure 5
Data were acquired with defocus only and radial filtering applied during IMOD tomogram reconstruction with the software default values. Scale bar: 100 nm. Tomographic slice thickness: 6.52 nm.

**Figure S5:**
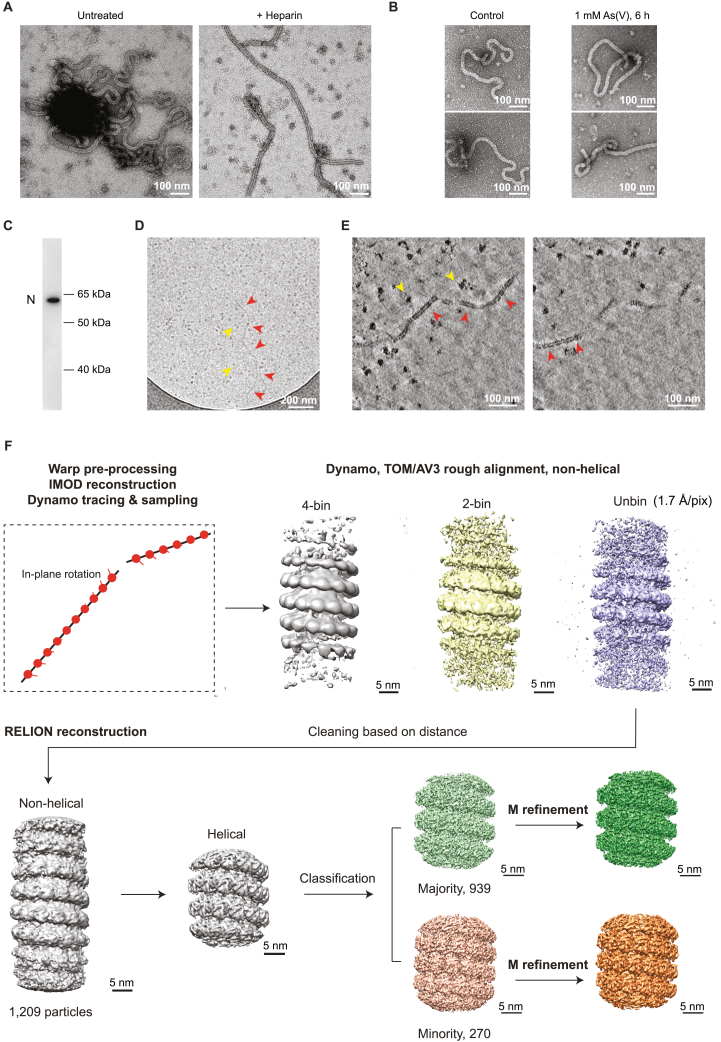
Structure determination of extracted MuV nucleocapsids, related to Figure 6 (A) Negative-staining TEM of nucleocapsids enriched in the 13,000 g pellet from cell lysates (left), and the effect of heparin addition on nucleocapsid morphology. The cells were stressed with 0.5 mM As(III) for 1 h. The heparin-straightened nucleocapsid sample was used for data acquisition and structural analysis in Figures 6 and S5D–S5F. (B) Negative-staining TEM of nucleocapsids enriched in the 13,000 g pellet from lysates of cells at the non-stress control or 6 h of 1 mM As(V) stress. (C) Western blot detects full-length MuV-N in the 13,000 g pellet from lysate of cells after 1 h of 0.5 mM As(III) stress. (D) Representative cryo-TEM image of extracted nucleocapsids (13,000 g pellet, red arrowheads) at intermediate magnification. Nearby ribosomes are labeled (yellow arrowheads). (E) Two 3.39 nm-thick tomographic slices of the nucleocapsid in (D), 12 nm apart in the z direction. Red arrowheads: straight segments of a long nucleocapsid used for structural analyses; yellow arrowheads: ribosomes. (F) Subtomogram averaging workflow for the extracted nucleocapsids from HeLa-MuV cells after ∼1 h of 0.5 mM As(III) stress. Frames in each tilt (20 tilt series in total) were aligned, averaged, and CTF-estimated in Warp (CTF, contrast transfer function). Averaged tilt series images were aligned and reconstructed into tomograms in IMOD. Alignments were imported into Warp for 3D-CTF estimation, tomogram reconstruction and application of a deconvolution filter. Nucleocapsids were traced manually in Dynamo. 1,209 center positions along the nucleocapsids were used to crop subtomograms with orientations assigned based on filament directions, with 30° in-plane rotation initially assigned to subsequent subtomograms. Template-free alignment was done in Dynamo at 4 times binning. Alignment results and average were used for subsequent processing of 2 times binned and unbinned subtomograms in TOM/AV3. No helical symmetry was applied at these stages. 3D refinement in RELION was first performed without applying symmetry until helical parameters could be determined in RELION. Helical reconstruction and classification were then done to separate subtomograms of different helical parameters. M refinement with symmetry expansion subsequently improved resolution and map quality. Except for the Dynamo alignment step, CTF-corrected subtomograms and 3D CTF models were all reconstructed in Warp. Overlapping particles were removed in intermediate steps and particle numbers used in each step are indicated. Also see STAR Methods.

To interpret the morphological transitions in the nucleocapsids, we first aimed to generate a structure for the authentic MuV nucleocapsids. Obtaining high-resolution structures of *Paramyxoviridae* family nucleocapsids has long been impeded by their curved and flexible nature. Available structures (detailed in Table S2) were enabled either by digestion or truncation of the flexible C-terminal region of the N protein to induce rigidity or by recombinant N protein expression that resulted in a ring shape or variable helical assemblies.^22^^,^^52^^,^^53^^,^^54^ Here, we isolated the authentic MuV nucleocapsids from stressed cells (Figures S5A–S5C). After 1 h of 0.5 mM As(III) treatment, the isolated nucleocapsids were curved, similar to their intracellular morphology at early time points of stress but straightened on addition of the RNA-mimicking molecule heparin (Figure S5A). This change in morphology was similar to the change in nucleocapsids seen inside cells at prolonged stress conditions (Figures 5C, 5D, and S4D). Interestingly, straight nucleocapsids present in VFs upon prolonged stress did not maintain their morphology once isolated and became curved (Figure S5B), suggesting that factors within their native viral condensate environment, such as increased local RNA levels (Figures 1E and 1F) possibly mimicked by the heparin addition *in vitro* or alterations in the interaction partners (Figure 5C insets), may contribute to the change in nucleocapsids from bent to straight conformations.

**Figure 6:**
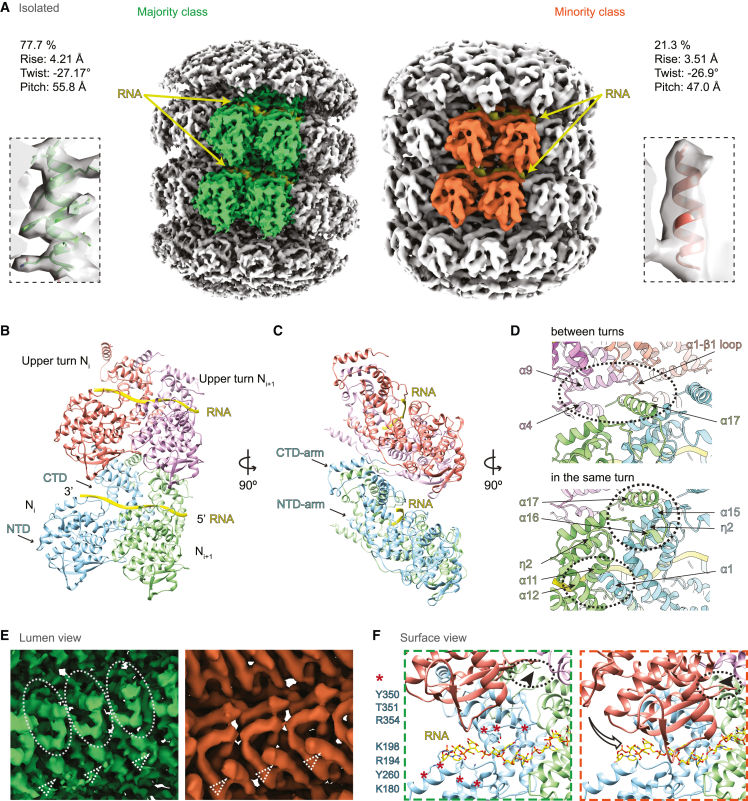
Structures of isolated MuV nucleocapsids reveal two packing modes with variable surface accessibility of RNA and C-terminal IDRs (A) Two classes of subtomogram averages of extracted nucleocapsids resolved to 4.5 Å (majority class) and 6.3 Å (minority class). Four subunits of each average are segmented to illustrate their different packing. RNA densities are colored in yellow. Zoomed-in views illustrate the quality of the map and fitted models. (B and C) Cartoon representation of the atomic model of MuV-N (amino acids 3–405) with RNA for four subunits of the majority class. (D) Subunit packing (colored as in B) between neighboring turns (upper panel) and within the same turn (lower panel). Circles: packing interfaces. (E) Lumen view comparison of the two classes in (A). CTD arms of neighboring subunits (green, circled) are not resolved in the minority class (orange). Dotted arrowheads: NTD-arms resolved in both maps. (F) Surface view comparison of the two classes (colored as in B). Key residues potentially responsible for RNA binding are labeled (red asterisks, listed on the left). Arrow in the orange frame indicates a shift of the upper subunit toward the lower turn RNA binding pocket in the minority class. Dotted circles indicate unoccupied space in the majority class (left) that becomes occupied in the minority class (right), which blocks access of the C terminus (arrowheads) to the nucleocapsid surface. See also Figures S5 and S6.

**Figure S6:**
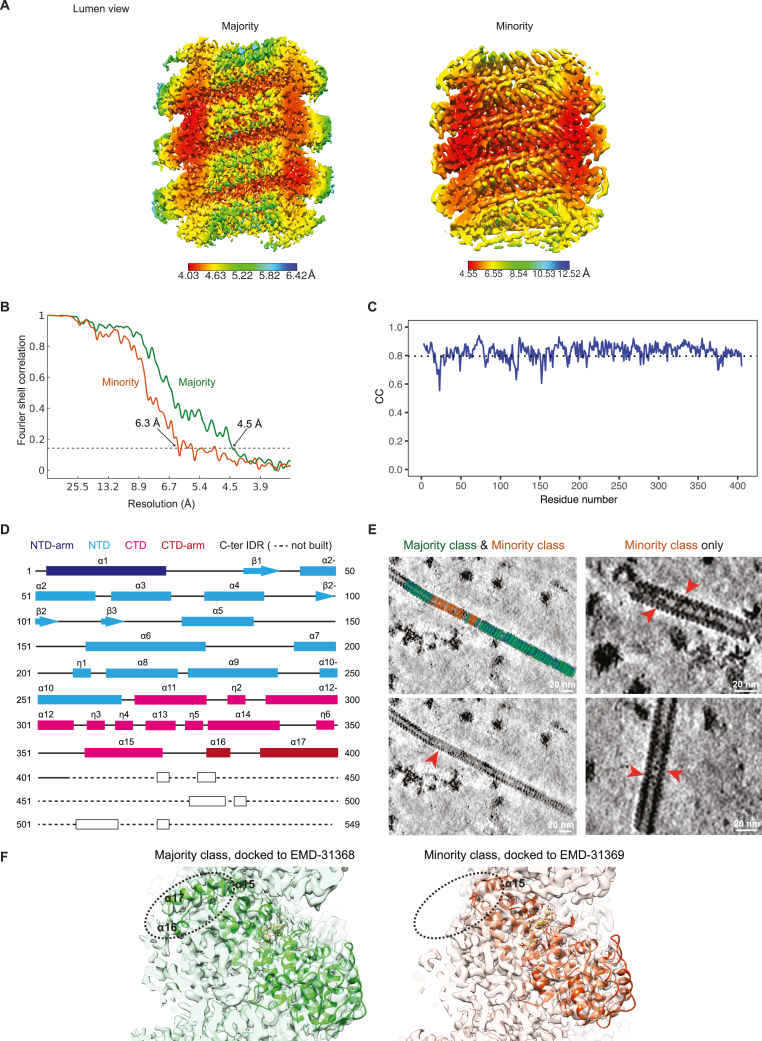
Structural analysis of the two classes in extracted nucleocapsids, related to Figure 6 (A) Local resolution maps for the majority and minority class averages. (B) Fourier shell correlation (FSC) plots calculated between the two independently refined half-sets for the majority and minority maps shown in (A). Global resolution is provided at the 0.143 criterion. (C) Plot of per residue cross correlation (CC) coefficients between the generated atomic model for N (amino acid 3–405) and the segmented subunit in the majority class map. Dotted line: average CC value of 0.80. (D) Illustration of the secondary structure of N. Secondary structure of the C-ter IDR was predicted with PSIPRED.^112^ (E) Mapping of two classes into tomograms. Red arrowheads indicate minority class mapped regions, showing densities in the nucleocapsid lumen, possibly representing the C-ter IDR of N protein in this conformation. (F) Rigid body fitting of the models generated in this study to the cryo-EM maps of helix-dense (EMD: 31368, green, similar to our majority class) and helix-hyper (EMD: 31369, orange, similar to our minority class) of the *in vitro* reconstituted nucleocapsids reported by Shan et al.^22^ The critical CTD-arm region (encircled, helices 16 and 17) is only resolved in one class (helix-dense by Shan et al., similar to our majority class), while it is missing in the other class in line with our results.

We obtained two maps from subtomogram averaging on the heparin-straightened nucleocapsids isolated from cells after 1 h of 0.5 mM As(III) stress (Figures 6A, S5D, and S5E). The two maps exhibited distinctly different helical parameters: a 5.58 nm long-pitch structure constituting the majority class (77.7%) was resolved to 4.5 Å and a 4.70 nm short-pitch class (21.3%) was resolved to 6.3 Å (Figures 6A, S5F, S6A, and S6B; Table S3). Excluding the most flexible C-terminal IDR of N (144 amino acids) that was not resolved in either map, we built an atomic model for the N protein into the high-resolution majority class based on a homologous structure from PIV5 nucleoprotein (PDB: 4XJN; Figures 6B, S6C, and S6D; Table S3; Video S4). The model showed that the viral RNA genome is accommodated at the nucleocapsid outer surface, supported by the existence of a clear density in the map, within a groove connecting the N-terminal domain (NTD) with the C-terminal domain (CTD) (Figures 6B, 6C, and S6D; RNA density in yellow, Figure 6A). The RNA packaging of MuV follows the “rule of six,” i.e., six nucleotides per N subunit,^53^ and is coordinated by conserved residues from a loop containing Tyr350, Thr351, Arg354 in the upper CTD, and loop and helices containing Lys198, Arg194, Tyr260, Lys180 from the lower NTD (Figure 6F). Interactions between subunits in consecutive turns of the nucleocapsid are mediated by the CTD-arm helix (a region succeeding the CTD), which inserts between the NTDs of two subunits in the upper turn (Figure 6D). Within the same turn, domain swapping between the NTD arms (a region preceding the NTD) and CTD arms of consecutive subunits formed extensive contacts at the nucleocapsid lumen (Figure 6D). Compared with the map of the majority class, density of this critical CTD-arm was missing in the map of the minority class indicating its high flexibility (Figure 6E), as only full-length protein was detected by western blot analysis (Figure S5C). Interestingly, both structural classes existed within the same isolated nucleocapsid (Figure S6E), in line with a study on MeV,^55^ and closely matching two recently reported high-resolution maps of recombinant MuV nucleocapsids^22^ (Figure S6F). Previous studies proposed a regulatory role of the C terminus of N in transcription and replication of *Paramyxoviridae*.^56^^,^^57^^,^^58^ Our two maps thus reflect the structural plasticity of N^10^^,^^22^^,^^51^^,^^59^ suggested to contribute to different functional outcomes: genome packaging to protect the viral genome from host antiviral factors versus genome accessibility to promote viral replication. Indeed, tighter packing of subunits in the minority class resulted in an 8.4 Å shift of a loop in the upper subunits toward the lower subunits, leading to less surface-exposed RNA binding pocket (Figure 6F; RNA density in yellow is buried in comparison with the majority class, Figure 6A). The tighter packing also restricted the space available for the flexible C-terminal IDR of N to extend to the nucleocapsid surface (Figures 6F and S6E). In contrast, the loosely packed structure of the majority class exhibits higher exposure of the RNA on the nucleocapsid surface and higher likelihood for the unresolved C-terminal IDR of N protein to extend and interact with partner proteins, i.e., P and L, at the nucleocapsid surface. Both factors would contribute to a conformation that can support viral transcription and replication.

**Figure 7:**
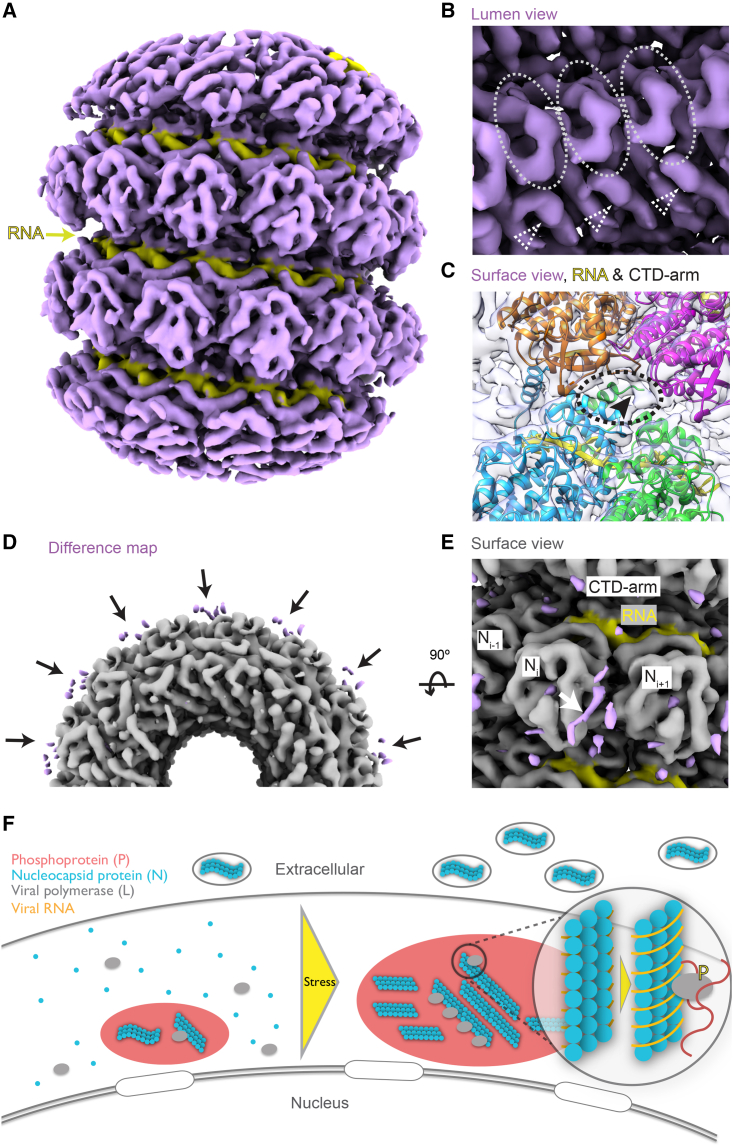
In-cell structure of MuV nucleocapsids at prolonged stress exhibits an RNA accessible state (A) Subtomogram average of the in-cell MuV nucleocapsids resolved to 6.5 Å. RNA densities in yellow. (B) Lumen view of the in-cell MuV nucleocapsid. CTD arms of three neighboring subunits are encircled and the NTD-arms are indicated with dotted triangles, similar to the majority class in Figure 6E. (C) Zoom-in surface view of the in-cell MuV nucleocapsid shows accessible RNA and CTD-arm (indicated with black arrowhead and the surrounding region with dotted circle). Subunits are docked and colored as in Figure 6F. (D and E) Cross-section and surface views of the difference map (purple) between the majority class in Figure 6A (gray) and the in-cell nucleocapsids. Densities in purple are only found in the in-cell map, indicating potential location of the C-terminal IDR at the interface between neighboring subunits. RNA density in yellow. (F) Proposed model for stress-induced activation of viral replication in phase-separated MuV factories. See also Figure S7.

Video S4 EM maps and structural models showing different conformations of the authentic MuV nucleocapsid, determined *ex* and *in situ*, related to Figures 6 and 7

### Nucleocapsids at prolonged stress adopt a replication accessible state

**Figure S7:**
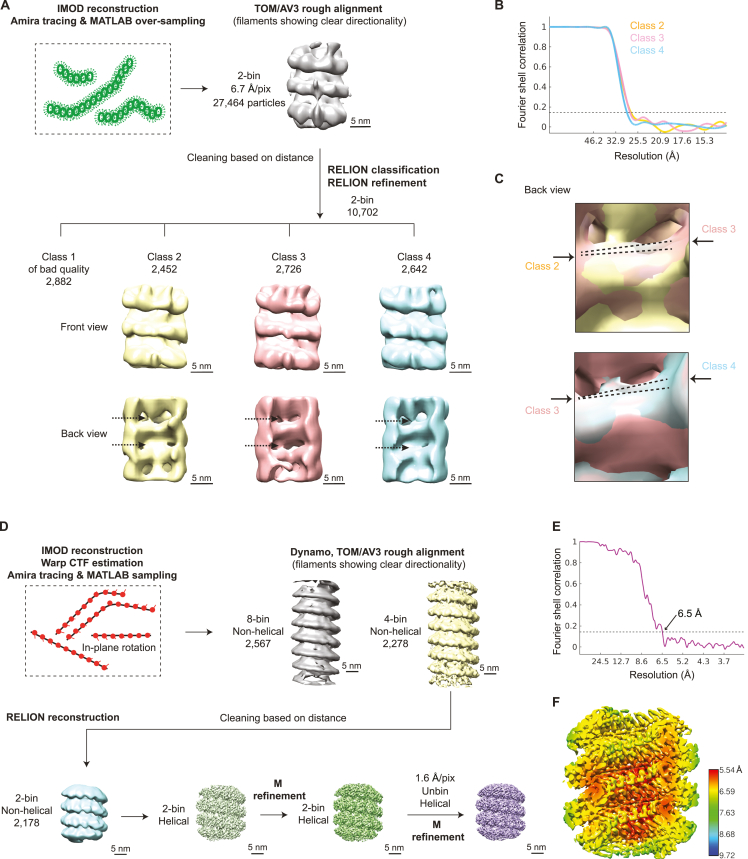
Subtomogram averaging for nucleocapsids *in situ*, related to Figure 7 (A) Subtomogram averaging workflow for curved nucleocapsids inside HeLa-MuV cells at 1 h of 0.5 mM As(III) stress, the same condition used for structure determination of isolated nucleocapsids. Nucleocapsids were traced by filament tracing in Amira and sampled with custom-made MATLAB scripts. An over-sampling strategy for particles at the nucleocapsid surface was used because the filaments are too curved to generate moderate or high resolution maps and to be averaged by helical reconstruction. After initial alignment in TOM/AV3, RELION classification resulted in three classes with mixed helical rises. The connecting densities between turns in back view (luminal, arrows) show high heterogeneity within each average, leading to difficulty in determination of exact helical parameters. Also see STAR Methods. (B) FSC plots for the class averages in (A), with the 0.143 criterion. (C) Map comparison between class averages in (A). The dotted lines qualitatively represent the different inclination of the helical turns in the compared classes. (D) Subtomogram averaging workflow for the straight nucleocapsids inside HeLa-MuV cells at 6 h of 30 μM As(III) mild stress and 1 mM As(V) acute stress. Particles from 6 tomograms at the two prolonged stress conditions were merged. Averaged tilt series images were CTF-estimated and reconstructed into tomograms in Warp with alignment files from IMOD, and denoised in Warp. Nucleocapsids were traced by filament tracing in Amira and sampled along the central lines with custom-made MATLAB scripts. Initial orientations of subtomograms were assigned based on filament directions with 30° in-plane rotation for subsequent subtomograms. Template-free alignment was done in Dynamo at 8 times binning. Alignment results and average were used for processing of 4 times binned subtomograms in TOM/AV3. No helical symmetry was applied at these stages. 3D refinement in RELION was first done at 2 times binning without applying symmetry, until helical parameters could be determined. Helical reconstruction was then done to improve the resolution. M refinement with helical symmetry subsequently improved the map quality. Helical reconstruction was finally done with unbinned particles. Classification trials resulted in classes with very similar helical parameters, and are not shown here. CTF-corrected subtomograms and 3D CTF models were all reconstructed in Warp. Overlapping particles were removed in intermediate steps, and particle numbers at each step are indicated. See also STAR Methods. (E) FSC plot for the final map shown in (D), with the 0.143 criterion. (F) Local resolution map for the final map shown in (D).

Although a recent study on *in vitro* reconstituted MuV nucleocapsids also reported existence of two distinct helical assemblies,^22^ similar to the structures we describe above (Figure S6F), the limited biological context of isolated nucleocapsid structures hinders the understanding of their relevance to functional states with respect to viral replication. With the structural models for the authentic MuV nucleocapsid at hand, we next examined the conformation of nucleocapsids in the cellular context. Their high curvature at early time points of stress (1 h, Figure 5) limited the possibility to refine the density to high resolution and resulted in maps at ∼30 Å only, showing class averages of mixed helical rises (Figures S7A–S7C) in line with non-uniform conformations observed in the isolated nucleocapsids at the same stress time point. At prolonged stress (6 h), we resolved the straight nucleocapsids to 6.5 Å (30 μM As(III) and 1 mM As(V); Figures 7A and S7D–S7F). Despite classification attempts, only a single class was obtained indicating overall conformational homogeneity in the intracellular straight nucleocapsids at prolonged stress. The atomic model generated for the loosely packed majority class of the isolated nucleocapsids fitted well into the map of the cellular nucleocapsids and could explain the majority of the density, showing the luminal CTD-arm (Figure 7B) and surface exposed RNA (Figures 7A and 7C; RNA density in yellow). However, a difference map calculated between the two similar maps of the isolated loosely packed majority class and the single class of cellular nucleocapsids visualized additional density at the nucleocapsid surface exclusively found in the in-cell map (Figures 7D and 7E) and which could potentially accommodate a short helix. This region was previously speculated to form the interaction interface between N and P, mediated by their disordered C termini.^10^^,^^58^ We propose that the additional density in the in-cell map could be part of the 144 amino acids long C-terminal IDR of N that becomes conformationally restricted due to its interaction with P under prolonged stress, consistent with our observation of abundant flexible densities near the nucleocapsids in the cellular tomograms (Figure 5C, right). Taken together, we conclude that in cells under prolonged stress, the nucleocapsids adopt a homogeneous conformation with a surface exposed viral genome that is likely to be more accessible for efficient transcription and replication by L.

## Discussion

Our study demonstrates how a combination of whole-cell proteomics and cryo-ET provides a powerful tool to probe molecular and structural aspects of viral replication in the context of biomolecular condensates inside cells and allows us to propose a mechanistic model for stress-mediated reactivation in persistent infection (Figure 7F). Using MuV as a model of negative-stranded RNA viruses, we show that persistent VFs in the form of dynamic condensates maintain low levels of viral replication and release that are not detrimental to the host. The released viruses are nevertheless contagious and can establish an infection in naive cultures within days. Stress to the host triggers posttranslational modification of P, likely by host factors, at its critical interaction interface with the viral RNA-dependent RNA polymerase. This can lead to increased partitioning of the polymerase into the VFs, as indicated by a reduction in its solubility and stabilization of a replication-competent complex under stress. A concomitant structural transition in the nucleocapsid, potentially induced by the change in the protein interaction network in the VFs, allows the viral genomic RNA to become more accessible to the polymerase. These events, accompanied by a concerted down-regulation of the host antiviral response, collectively provide an environment that supports upregulation of viral replication under cellular stress.

An interplay between viral replication and cellular stress has been documented for many viruses in the acute phase of infection following cell entry. MuV and rabies virus infection lead to the formation of stress granule via activation of the protein kinase R (PKR) stress pathway by the viral RNAs.^60^^,^^61^ In contrast, MeV can interfere with stress granule formation^62^ or others, like Zika virus, take advantage of key stress granule proteins or the stress response pathways (e.g., G3BP1) to promote viral gene expression.^63^ Furthermore, acute phase replication of influenza virus is enhanced in cells exposed to arsenite.^64^ However, little is known about how stress affects viruses at the chronic infection phase. Exceptions are studies of social stress reported to cause reactivation of latent DNA virus, e.g., HSV-1,^2^ and of oxidative stress leading to reactivation of HIV-1.^3^ Our proposed stress-reactivation model for MuV persistent infection may thus represent generic molecular mechanisms for reactivation of chronic RNA viruses. First, phosphorylation of the viral P protein may induce a cascade of changes in the viral replication machinery that accelerates transcription and replication. The intracellular levels of many kinases and phosphatases changed under stress (STAR Methods). However, the enzymes responsible for P phosphorylation^65^^,^^66^ need further investigation, with changes in the activity of the enzymes considered alongside their abundance. Second, we observed that the magnitude of increase in replication activity correlated with the severity of stress: genomic RNA level under mild As(III) stress was slightly suppressed until 6 h before showing a dramatic upregulation at 24 h, reminiscent of acute phase infection where transcription is prioritized to generate more material (e.g., polymerase and nucleocapsid protein) required for replication.^67^ Third, the cross-talk between the stress response and cytokine signaling, exemplified by the downregulation of IFN signaling factors, such as JAK1^68^ further permits viral replication. We observed that viral budding oscillated in both the non-stressed and stressed cells over 24 h. In line with this, feedback mechanisms must exist to maintain a long-term balance between the MuV and the host. Finally, the increase in virion release may result in a new infection or slow progression of severe chronic diseases.^6^^,^^9^

By integrating quantitative light microscopy, hexanediol solubilization, and transient expression of recombinant viral proteins for live imaging, we identified MuV factories as dynamic condensates that coarsen under stress. Cryo-ET further showed increased molecular crowding within the coarsened condensates. Enrichment of the viral genome-containing nucleocapsids along with the replication machinery—as shown by quantitative proteomics—within the condensates provides a mechanism to boost viral replication. A similar function was exemplified by the enhanced MeV genome encapsidation within droplets reconstituted *in vitro* with N and P proteins and RNA, compared with the dilute phase.^69^ On the other hand, the condensate-mediated MuV replication compartment can shield viral proteins and RNA from host antiviral factors by preventing for example induction of IFN effectors, or alternatively, by sequestering IFN mediators into the condensate to block their downstream antiviral effects; our proteomics data showed that V protein implicated in immunomodulation and the host JAK1 exhibit a marked decrease in solubility under stress (Figures 4B, S3D, and S3E; STAR Methods). Indeed, in cells persistently infected by MuV or its family member MeV, the IFN signaling pathway effector protein oligo-2’,5’-adenylate synthetase as part of the host immune response is reported to be suppressed.^27^ This is thought to be mediated by the V protein that directly disrupts the formation of the STAT1-STAT2 complex^70^ or the STAT1, receptor-activated C kinase (RACK1) and IFN receptor complex.^50^

Unlike previous reports on aging of recombinant MeV condensates,^14^ our study revealed no detectable change in the dynamic properties of authentic MuV factories associated with their increased size under stress, as probed by the hexanediol solubilization and FRAP assays. The formation of large VFs was observed not only in patient biopsies associated with chronic MuV infection but also during its acute infection in culture models.^45^ Prohibiting the formation of large factories using a dynein inhibitor during MeV infection resulted in significantly decreased viral titers.^14^ VF coarsening could therefore represent an essential stage in the viral life cycle, regulating viral replication, assembly, and budding. Interestingly, a recent study highlights the detrimental effect of VF solidification in human respiratory syncytial virus on replication.^71^ Thus, the link between the size of VFs, their properties, and viral replication is worth exploring in diverse viruses. We identified MuV P protein as a driving force for condensate formation and that it can recruit N protein. Consistent with these observations are previous findings in MeV showing that the interaction between the C termini of N and P is critical for their phase separation.^14^ Although the C-terminal sequences of N and P in MeV and MuV are distinct, both appear to be disordered. The rabies virus P protein was also found to be critical for phase separation via its dimerization domain and the flanking IDR.^15^ In vesicular stomatitis virus, N, P, and L proteins are all required for formation of condensates, whereas replication activity is not needed.^16^ In SARS-CoV-2, region-specific genomic RNA^20^ and the membrane protein^21^ can independently induce phase separation of its N protein, forming annular structures in which the membrane protein coats the outside of an N + RNA condensate. Even the distant HIV-1 was recently shown to utilize phase separation of nucleocapsid as a condensation platform to aid viral assembly together with RNA.^17^^,^^18^ This further leads us to speculate that in our MuV model, the increased viral transcripts and genomic RNA levels under stress may modulate the phase behavior of the viral condensates; although we show that an increase in genomic RNA levels in the condensates coincides with their coarsening, it remains unknown how viral transcripts may distinctively affect the properties and behavior of the VFs. Our cryo-ET data show that MuV VFs largely exclude ribosomes. Therefore, viral transcripts must be exported to their periphery for translation, as observed for rabies VFs.^61^ Phosphorylation of viral proteins adds another layer to the regulation of condensate properties. This was reported for MeV^14^ and SARS-CoV-2.^72^ In our MuV model, a phospho-mutant on the IDR of P protein did not lead to apparent changes in its partitioning into VFs, although it had a major effect on the interaction of the replication machinery with the genome-harboring nucleocapsid. P can be phosphorylated on multiple sites^73^ and the combinatorial effect of its phosphorylation states on condensation should be more systematically investigated. Altogether, these studies reveal that RNA viruses evolved multiple mechanisms to ubiquitously form condensate-mediated viral replication factories.

On the structural level, MuV nucleocapsids are templates for both viral transcription and replication. Their conformational flexibility has been reported for other contagious viruses with negative-stranded RNA genomes such as MeV^59^^,^^74^ and respiratory syncytial virus^75^ and hypothesized to contribute to regulation of the balance between transcription and replication.^59^ A previous study on MeV shows that two distinct helical assemblies of the nucleocapsids exist in released virions,^55^ but their functional relevance was not addressed due to limited resolution of the structures. In this study, we identified two conformations that coexist in the authentic MuV nucleocapsids isolated from cells after 1 h of cellular stress. These differed in the apparent flexibility of the CTD-arm region of N protein and surface exposure of the viral genome. The ratio of the two conformations may therefore be important for fine-tuning viral activity at persistent infection and for reactivation under stress. Interestingly, our in-cell map of nucleocapsids from prolonged stress conditions resolved to high detail represents a genome-accessible conformation, in line with increased viral transcription and replication detected in our PCR quantification and RNA FISH assay. Additional densities on the nucleocapsid surface revealed in this in-cell map in comparison with the isolated nucleocapsids are suggested to be the C terminus of N or its partner protein P, consistent with previous computational simulations^58^ or low-resolution structural models,^10^ further supporting active transcriptional/replication state of nucleocapsids under stress. Finally, the straight morphology of MuV nucleocapsids observed in our data mimics their appearance in chronic myositis, providing a link between these nucleocapsid conformations resolved in a culture model to human pathology.

In summary, our integrated structural cell biology study illustrates that stress-induced subtle changes in viral protein interaction networks and fine structural changes in the nucleocapsid, potentially amplified by the specialized condensate environment of the VFs that further concentrate the viral components, seem sufficient to disturb the delicate balance between the virus and host cell during persistent infection. It is tempting to speculate that such molecular switches induced by stress may have broad implications across a diverse family of viruses that establish chronic infection in the human host and for the diseases they elicit.

### Limitations of the study

Our study focused on how exogenous stress to the host may constitute a switch that induces changes in MuV factories to upregulate viral replication in a model of a HeLa cell culture. The questions of whether such events take place in the complex context of a human patient and whether the observed modest changes contribute to the development of pathology represent a major challenge. Our study provides first handles on the critical molecular components to be targeted; for example, further work aimed at the identification of the kinases and phosphatases responsible for the modulation of P phosphorylation levels and sites under stress may assist in intervention of MuV persistent infection and related diseases. Furthermore, the exact sequence of molecular events following stress that lead to changes in the nucleocapsid structure remains to be elucidated. Our data suggest that the condensate environment may affect the conformational state of the nucleocapsids. On the one hand, it is possible that the stress-induced morphological change in the nucleocapsid is associated with increased RNA levels in the condensates leading to charge or steric effects. On the other hand, the molecular grammar of the interacting proteins, especially of their IDRs that form critical interaction interfaces with other IDRs or structured domains in the complex mixture of viral proteins, remains to be systematically probed in higher detail. Follow-up *in vitro* reconstitution with engineered and reduced viral components will help to elucidate the magnitude of the different effects. Finally, the mechanism and functional implications of the observed spatial proximity between the MuV factories and stress granules, not discussed in this study, are intriguing avenues to explore in the future with genetically engineered viruses and cell lines to understand factors mediating their potential association.

## STAR★Methods

### Key resources table

**Table:** 

REAGENT or RESOURCE	SOURCE	IDENTIFIER
**Antibodies**		

Mouse monoclonal anti-MuV-N protein (clone 8H4)	Abcam	Cat#ab9876; RRID:AB_296684
Goat anti-mouse IgG secondary antibody Alexa Fluor 488 (highly cross-adsorbed)	Invitrogen	Cat#A-11029; RRID:AB_2534088
Goat anti-mouse IgG secondary antibody Alexa Fluor plus 647 (highly cross-adsorbed)	Invitrogen	Cat#A-32728; RRID:AB_2633277
Mouse monoclonal anti-GAPDH (clone 6C5)	Santa Cruz	Cat#sc-32233; RRID:AB_627679
Goat anti-mouse IgG (H/L):HRP (multi species absorbed)	Bio-Rad	Cat#STAR117P; RRID:AB_323839

**Bacterial and virus strains**		

Mumps virus Enders strain	Accidental infection in the HeLa cell culture	GenBank: GU980052 Single nucleotide variations from Enders strain are reported below

**Chemicals, peptides, and recombinant proteins**		

Sodium arsenite	Sigma-Aldrich	S7400; CAS: 7784-46-5
Potassium arsenate	Sigma-Aldrich	A6631; CAS: 7784-41-0
1,6-Hexanediol	Sigma-Aldrich	240117; CAS: 629-11-8
ProLong Gold Antifade Reagent with DAPI	Cell Signaling Technology	Cat# 8961
Oligo d(T)_23_ primer	New England Biolabs	Cat#S1327S
EDTA-free protease inhibitor cocktail	Roche	04693132001
PhosSTOP Phosphatase inhibitor cocktail	Roche	4906845001
Benzonase Nuclease, ultrapure	Millipore	E8263; CAS: 9025-65-4
Ribonuclease inhibitor	Sigma-Aldrich	R1158
Heparin sodium	Sigma-Aldrich	H3149; 9041-08-1
Protein-A/Gold, EM-grade 10 nm	Electron Microscopy Sciences	Cat#50-281-97
1-μm crimson beads (FluoSpheres carboxylate-modified microspheres, 625/645)	Invitrogen	Cat#F8816

**Critical commercial assays**		

jetPRIME Transfection Reagent	Polyplus	Ref: 101000027
RNeasy Micro Kit	Qiagen	Cat#74004
ProtoScript II Reverse Transcriptase Kit	New England Biolabs	Cat#M0368
TaqPath qPCR Master Mix, CG	Applied Biosystems	Cat#A15297
RNA Nano 6000 Assay Kit	Agilent Technologies	Cat#5067-1511
NEBNext Ultra II Directional RNA Library Prep Kit for Illumina	New England Biolabs	Cat#E7760
SPRIselect beads	Beckman Coulter	B23318
DNA High Sensitivity kit	Agilent Technologies	5067-4626
Qubit dsDNA High Sensitivity kit	Invitrogen	Cat#Q32851
Bradford Protein Assay Dye	Bio-Rad	Cat#5000006
Stellaris RNA FISH Wash Buffer A	Biosearch Technologies	Cat#SMF-WA1-60
Stellaris RNA FISH Hybridization Buffer	Biosearch Technologies	Cat#SMF-HB1-10
Stellaris RNA FISH Wash Buffer B	Biosearch Technologies	Cat#SMF-WB1-20
GFP-Trap magnetic agarose	ChromoTek	Cat#gtma-20

**Deposited data**		

PIV5 nucleocapsid-RNA complex	Alayyoubi et al.^52^	PDB: 4XJN
PIV5 L-P complex	Abdella et al.^46^	PDB: 6V85
Cryo-EM map of PIV5 L-P complex	Abdella et al.^46^	EMD: 21095
Cryo-ET of viral factory at non-stressed condition	This study	EMD: 13165
Cryo-ET of Viral factory at 1 h of 0.5mM As(III) stress	This study	EMD: 13166
Cryo-ET of Viral factory at 6 h of 30 μM As(III) stress	This study	EMD: 13167
Cryo-EM map for isolated nucleocapsids, majority class	This study	EMD: 13133
Cryo-EM map for isolated nucleocapsids, minority class	This study	EMD: 13136
Cryo-EM map for straight cellular nucleocapsids	This study	EMD: 13137
Model for isolated nucleocapsids, majority class	This study	PDB: 7OZR
Raw micrographs for isolated nucleocapsids	This study	EMPIAR: 10751
Raw mass spectrometry data	This study	ProteomeXchange: PXD026799
Reference proteome sequence for *Homo sapien*	Uniprot	Uniprot: UP000005640
Processed mass spectrometry data	This study	Mendeley Data: https://doi.org/10.17632/4wzny9kwmn.1
Single nucleotide variations of the MuV strain	This study	Mendeley Data: https://doi.org/10.17632/4wzny9kwmn.1
Reference genome NCBI	Young et al.^76^	GenBank: GU980052

**Experimental models: Cell lines**		

HeLa: G3BP1-mCherry BAC in HeLa Kyoto cell line	Guillen-Boixet et al.^28^	MCB_ky_7510 (C-terminal mCherry tag; FAC sorted)
HeLa-MuV: G3BP1-mCherry BAC in HeLa Kyoto cell line persistently infected by mumps virus	Parental cell line from Guillen-Boixet et al.^28^	N/A
U2OS: G3BP1-mCherry BAC cell line	Parental cell line from Guillen-Boixet et al.^28^	N/A

**Recombinant DNA**		

pcDNA3.1-MuV-P	This study (synthesized by GeneArt/Life Technologies)	N/A
pcDNA3.1-MuV-P-EGFP	This study	N/A
pcDNA3.1-MuV-N	This study (synthesized by GeneArt/Life Technologies)	N/A
pcDNA3.1-MuV-P-EGFP (P_deficient)	This study (synthesized by GenScript)	N/A

**Software and algorithms**		

SerialEM	Mastronarde^77^	https://bio3d.colorado.edu/SerialEM/
Dose-symmetric tomography acquisition scheme	Hagen et al.^78^	N/A
IMOD package	Kremer et al.^79^	https://bio3d.colorado.edu/imod/
3DCT 2.2.2	Arnold et al.^80^	https://3dct.semper.space/
Amira 6.7 and 2019.4	ThermoFisher Scientific	https://www.fei.com/software/amira-release-notes/
Warp 1.0.9	Tegunov and Cramer^81^	http://www.warpem.com
M 1.0.9	Tegunov et al.^24^	http://www.warpem.com
Matlab R2019a	MathWorks	https://www.mathworks.com
Dynamo 1.1.401	Castano-Diez et al.^82^	https://wiki.dynamo.biozentrum.unibas.ch
TOM	Nickell et al.^83^	N/A
AV3	Forster and Hegerl^84^	N/A
RELION 3.0 and 3.1	Zivanov et al.^85^	https://github.com/3dem/relion
I-TASSER	Roy et al.^86^	https://zhanglab.dcmb.med.umich.edu/I-TASSER/
Coot 0.9	Emsley et al.^87^	https://www2.mrc-lmb.cam.ac.uk/personal/pemsley/coot/
Phenix 1.18-3845	Liebschner et al.^88^	https://phenix-online.org
Chimera 1.13.1	Pettersen et al.^89^	https://www.cgl.ucsf.edu/chimera/
ChimeraX 1.1.1	Pettersen et al.^90^	https://www.cgl.ucsf.edu/chimerax/
Fiji 2.1.0	Schindelin et al.^91^	https://imagej.net/software/fiji/
FRAPAnalyser 2.0	N/A	https://github.com/ssgpers/FRAPAnalyser
Imaris 9.5.1	Oxford Instruments Group	https://imaris.oxinst.com
Image Lab 6.1	Bio-Rad	https://www.bio-rad.com
R 3.6.1	R	https://www.r-project.org
RStudio 1.4.1103	RStudio	https://www.rstudio.com
Prism 6.0c	GraphPad	https://www.graphpad.com
IUPred2A	Meszaros et al.^92^	https://iupred2a.elte.hu/
PSIPRED	Jones^93^	http://bioinf.cs.ucl.ac.uk/psipred/
BWA-MEM 0.7.17-r1188	N/A	https://github.com/lh3/bwa
Picard tool 2.9.0	Broad Institute of MIT and Harvard	https://broadinstitute.github.io/picard
FreeBayes 1.1.0-3	N/A	https://github.com/freebayes/freebayes
isobarQuant	Franken et al.^94^	https://github.com/protcode/isob
Mascot 2.4	Matrix Science	https://www.matrixscience.com
vsn	Huber et al.^95^	https://bioconductor.org/packages/release/bioc/html/vsn.html
limma	Ritchie et al.^96^	https://bioconductor.org/packages/3.14/bioc/html/limma.html
clusterProfiler	Yu et al.^97^	https://bioconductor.org/packages/3.14/bioc/html/clusterProfiler.html
3D-Unet DeePiCt	de Teresa et al.^98^	https://github.com/irenedet/3d-unet/tree/7bc343971bdb818c5de90570b83731c8d77cde04
AlphaFold2	Evans et al.^48^	https://github.com/deepmind/alphafold

### Resource availability

#### Lead contact

Further information and requests for resources and reagents should be directed to and will be fulfilled by the lead contact, Julia Mahamid (julia.mahamid@embl.de).

#### Materials availability

All unique materials and reagents generated in this study are available from the lead contact with a completed material transfer agreement.

### Experimental model and subject details

#### Cell culture

The HeLa cell line stably expressing mCherry-G3BP1 by bacterial artificial chromosome (BAC) tagging^99^ was previously described.^28^ Cells were cultured in Dulbecco's modified Eagle's medium (DMEM, high glucose, GlutaMAX Supplement, Gibco) supplemented with 10% fetal bovine serum (FBS), 50 U/ml Penicilin-Streptomycin (P/S) and 2 μg/ml Blasticidin S (Gibco), and maintained using standard procedures. We accidently encountered the persistent MuV infection (strain Enders, for single nucleotide variations see Key Resources Table) in our cell culture (HeLa-MuV cells). Persistent infection in a mCherry-G3BP1 BAC U2OS cell line^28^ was established as reported previously and cultured in the same manner as HeLa-MuV cells.^27^ Cell lines have not been authenticated.

#### Plasmids

The plasmids encoding MuV untagged N or EGFP (enhanced green fluorescent protein)-tagged P were synthesized by GeneArt Gene Synthesis (Thermo Fisher Scientific), both in a pcDNA3.1 vector. The EGFP gene was inserted at the N-terminus of P gene. Phospho-deficient mutant (S292A, S294A, T298A, S301A; all four mutated, P_deficient) plasmid was synthesized by GenScript in a pcDNA3.1 vector with the EGFP gene inserted at the N-terminus of the P gene.

### Method details

#### Proliferation test

For testing the effect of persistent infection on the proliferation of HeLa cells, non-infected and persistently infected HeLa (HeLa-MuV) mCherry-G3BP1 BAC cells were seeded as 24 replicates for each line on 24-well plates at a density of 5 × 10^6^ cells/well. Cell numbers were counted by collecting cells in 6 wells (as 6 replicates) for each line every 24 hours for 4 consecutive days. Trypan Blue Dye (Bio-Rad) staining was performed and numbers of viable cells were read from a TC20 automated cell counter (Bio-Rad).

#### Stress treatment and immunofluorescence (IF) microscopy

For IF microscopy, HeLa-MuV cells (mCherry-G3BP1) were seeded on 35-mm dishes with a glass bottom the day before stress treatment. Cells were stressed by replacing the culture medium with that containing either 0.5 mM sodium arsenite (referred to as As(III)) for up to 3 h, 1 mM potassium arsenate (referred to as As(V)) for up to 6 h, 30 μM As(III) for up to 24 h, or transferring the cell culture dishes to an incubator set to 43 °C for 1 h for heat shock. Samples of all time points under a particular stress condition were fixed at once with 4% paraformaldehyde (PFA) pH 7.4 in phosphate-buffered saline (PBS: 2.67 mM KCl, 1.5 mM KH_2_PO_4_, 137 mM NaCl, and 8.1 mM NaH_2_PO_4_, pH 7.4) for 10 min, after rinsing twice with PBS. Samples were rinsed with cold PBS and permeabilized with 0.2% Trion X-100 for another 10 min. Samples were blocked with 1% bovine serum albumin (BSA) for 30 min, incubated with mouse monoclonal antibody against MuV N protein (1:200; clone 8H4; Abcam, ab9876) diluted in 1% BSA for 1 h at room temperature and then with goat anti-mouse IgG secondary antibody Alexa Fluor 488 (1:2,000; highly cross-adsorbed; Invitrogen, A-11029). The samples were finally immersed in ProLong Gold Antifade Mountant formulated with the DNA stain DAPI (Thermo Fisher Scientific) and sealed. Confocal imaging was performed on a Zeiss LSM780 inverted microscope with a 63× 1.40 NA oil immersion objective and a GaAsP spectral detector. Settings were kept the same for imaging samples of all time points in one experiment. Z-stack images were segmented using the Imaris software (Oxford Instruments) using the Surface module. Surfaces of viral factories were generated by absolute intensity thresholding with a fixed value for the anti-N staining signal in each dataset (values optimized for different datasets) and surfaces of nuclei by background subtraction auto-thresholding of the DAPI staining signal, separately. Distances between viral factories and nuclei were calculated for each image stack with MATLAB (MathWorks) scripts to assign viral factories to individual cells. Volumes and numbers of viral factories per cell were then used for generating density plots with RStudio scripts. U2OS cells persistently infected with MuV were treated with 1 mM As(V) for 6 h and fixed and immunostained in the same way. For U2OS cells, central slice images were acquired. 2D segmentation was also done using the surface module in Imaris software and quantification done with MATLAB and RStudio scripts similarly.

All light microscopy figures were prepared with FIJI.^91^ Maximum intensity projected images are shown for Z-stack images unless stated otherwise.

#### Infection assay

To test if the MuV virions released during persistent infection are infectious, naïve HeLa mCherry-G3BP1 cells (not infected by MuV) were used for an infection assay. One day before the assay, naïve HeLa mCherry-G3BP1 cells were seeded on one 8-well slide (Ibidi, μ-Slide) at a concentration aimed to attain 80-90% confluency on the day of infection. Virions were collected from the culture medium of HeLa-MuV cells after removing cell debris by centrifugation at 2200 *g*, 4 °C for 15 min. The original virion stock was serially diluted to 10^-1^, 10^-2^, 10^-3^, 10^-4^, 10^-5^, 10^-6^ solutions with DMEM medium (no FBS or P/S). After removing the culture medium of the pre-seeded naïve HeLa mCherry-G3BP1 cells and rinsing the cells twice with PBS, 100 μL of the serially diluted solutions and the original virion stock were added separately to each well and incubate for 2 h with shaking every 30 min. After replacing the infection solution with complete culture medium, cells were cultured for 48 h, fixed and processed for viral factory formation by immunostaining as described above.

For testing the effect of stress on infectious virion production, at 24 h after cell seeding, half of the volume of the cell culture medium (2.5 ml per dish) was collected from HeLa cells persistently infected with MuV at the 0 h timepoint, and then the second half was collected from the same dishes at 6 h of non-stress control or 30 μM As(III) stress (sodium arsenite being directly added to the remaining culture medium in flasks). Cell numbers were counted at 6 h for both stress and control conditions and determined to be of similar levels. PEG precipitation and resuspension of virions (with DMEM, no FBS or P/S) was performed as for western blot experiments described below in order to minimize residual arsenite. Infection assays were then performed as described above. Imaging was performed on an Olympus FV3000 confocal microscope with a 60 × 1.3 NA silicone oil immersion objective and GaAsP PMT detectors. Z-stack images were segmented using the Imaris software using the Surface module as described above. The sum volume of viral factories per image was normalized to the number of cells per image (approximated by the number of nuclei) and fold change of stress over control conditions for each time point was calculated and plotted in RStudio.

#### Hexanediol treatment

Hexanediol treatment was done for assessing the properties of the authentic viral factories at 1 mM As(V) stress conditions. Culture medium was replaced with medium containing 1 mM As(V) as before for 1 h or 6 h to induce stress. Together with the unstressed cell samples as 0 h control, cells were either immediately fixed as control for each time point, or incubated with 3.5% 1,6-hexanediol (Sigma-Aldrich) or water for 15 to 30 min and then fixed. Immunostaining and imaging were done as described above. Central slice images were acquired. 2D segmentation was similarly done using the surface module in Imaris software. The number of viral factories larger than 2 μm in diameter (assuming a sphere) per cell was counted for each condition.

#### Transfection

Transfection of naïve HeLa mCherry-G3BP1 cells (not infected by MuV) was done with the untagged N and EGFP-tagged P plasmids. Transfection was done on pre-seeded HeLa cells (about 70% confluency) with the jetPRIME Transfection Reagent (Polyplus) following standard protocol. At 48 h after transfection, cells were fixed and immunostained against MuV N protein as described above. Imaging was performed on a PerkinElmer spinning disk confocal microscope with a 63 × 1.40 NA oil immersion objective and a Hamamatsu EMCCD camera. Central slice images were acquired.

In order to monitor the dynamics of MuV factories in HeLa-MuV cells or analyze the condensation of P protein in a close to native viral infection system, EGFP-P plasmids (wildtype: P_WT; or phospho-deficient mutant: P_deficient) were transfected into HeLa-MuV cells as described above. At 24 h after transfection, cells were either fixed and immunostained against MuV N protein as described above, or used for time-lapse imaging or fluorescence recovery after photobleaching experiments detailed below. Imaging was performed on Olympus FV3000 confocal microscope with a 60 × 1.3 NA silicone oil immersion objective and GaAsP PMT detectors. Partition coefficient of EGFP-P (WT or mutant) into viral factories was quantified according to Wang et al.^13^ using the same FIJI script. Specifically, maximum noise level was optimized based on the images and kept the same for analysis of the entire dataset. DAPI staining (ProLong Gold Antifade Reagent with DAPI; Cell Signaling Technology) and G3BP1-mCherry channels were used for determining the nuclear and cytoplasmic regions. Cells wherein EGFP-P condensates were not detected by the algorithm were not included for calculation of partition coefficient (here defined as a ratio of mean intensity of EGFP-P in condensates to that outside the condensates in cytoplasmic regions per cell). Background intensity was measured as mean intensity of regions outside of cells per image and subtracted (average intensity of three regions of 15-30 square pixels per image). Plots were generated with RStudio.

#### Time-lapse imaging

HeLa-MuV cells were transfected with the EGFP-P (WT) plasmid to measure the dynamics of MuV factories. At 24 hours post transfection, cells (seeded on 35-mm Ibidi dishes) were treated with culture medium containing 1 mM As(V) or 30 μM As(III) and immediately transferred to Olympus FV3000 confocal light microscope with a 60 × 1.3 NA silicone oil immersion objective and GaAsP PMT detectors for time-lapse imaging. Z-stack images of two channels (mCherry-G3BP1 and EGFP-P) were taken every 2 min within 3 hours of stress treatment.

#### Fluorescence recovery after photobleaching (FRAP)

As for the time-lapse imaging, HeLa-MuV cells transfected with EGFP-P_WT were used for the FRAP experiments. Cells were seeded on 35-mm Ibidi dishes and EGFP-P signal partitioning within MuV factories at the non-stressed condition (control) or up to 6 hours after the start of stress (1 mM As(V) or 30 μM As(III) treatment) was photo-bleached. Experiments were carried out using Olympus FV3000 confocal microscope with a 60 × 1.3 NA silicone oil immersion objective and GaAsP PMT detectors. A region of 15×10 pixels (∼3.11 μm × 2.07 μm) was selected within a condensate and bleached with 30-50% of a 20 mW 488 nm laser and a 16 msec dwell time for 3 times. The fluorescence intensity was recorded for 10 frames prior to the bleach and the recovery of fluorescence was recorded at 1 sec/frame for 150-300 sec.

Analysis of FRAP measurements was done with FIJI and FRAPAnalyser 2.0 (developed in the University of Luxembourg; https://github.com/ssgpers/FRAPAnalyser). Normalization was done with the ‘double normalization method’.^100^ First, the fluorescence intensity of the bleached region (I_frap_) within the condensate and that of the reference region (I_ref_; the total condensate intensity) were subtracted for background fluorescence (I_back_; outside of cells or far from the condensate regions inside cells) and scaled relative to their average prebleach intensity (also background subtracted) separately. Secondly, the correction was done by dividing the intensity values in FRAP region by those in reference region. Then the recovery was fitted with the normalized fluorescence data as a function of time to the single or double exponential equations for each region separately. Nelder-Mead (simplex) and Levenberg-Marquardt (gradient) methods were tested with the one giving better fitting of the data presented. The halftime (t_1/2_) and mobile fraction of the recovery were derived from fitting of each curve. The immobile fractions were calculated from the equation: Immobile Fraction = 1 – Mobile Fraction. Statistical analysis and plotting were carried out with GraphPad Prism 6.0c.

#### Quantitative PCR

0.5 to 1 million cells per condition and time point were used for isolating total RNA. The extraction was performed according to manufacturer’s instructions using the RNeasy Micro Kit (Qiagen). Strand-specific reverse transcription of genomic RNA (gRNA), anti-genomic RNA (cRNA) and mRNA was respectively performed with the 3’-UTR genomic forward primer, L-gene antigenomic reverse primer (Table S1), and oligo d(T)_23_ primer (New England Biolabs) for transcripts. For quantitative comparison between samples, cDNA was also generated for the cellular control gene RNase P using a gene specific reverse primer (Table S1). 1 μg of RNA was used to generate specific cDNAs for each condition, time point and target gene separately. Synthesis was performed according to manufacturer’s instructions using the ProtoScript II Reverse Transcriptase Kit (New England Biolabs).

Real-time quantitative PCR (qPCR) assay was performed using TaqPath qPCR Master Mix, CG (Thermo Fisher Scientific), target-specific primers and Taqman probes (Table S1). 20× primer probe mixes of each target gene were prepared with 150 nM forward primers, 150 nM reverse primers and 100 nM Taqman probes. Genome, and antigenomic cDNAs were mixed with the cellular control gene RNase P cDNA for each condition and time point at a dilution of 1:10. Transcript cDNAs generated with the oligo d(T)_23_ primer were diluted at 1:10 for each condition and time point. The qPCR was set up in 10 μl total mixture comprised of 5 μl Master Mix (2×), 0.5 μl N-gene primer-probe mix, 0.5 μl P-gene primer-probe mix, 0.5 μl F-gene primer-probe mix, 0.5 μl RNaseP control gene primer-probe mix, 2 μl H_2_O, and 1 μl cDNA mixes (1:10 dilution). For each plate, standard curves were generated at a dilution range of 1:10 to 1:10,000 with the non-treated control condition of each biological replica. qPCR was performed using the Roche LightCycler96 cycling conditions: 95 ºC for 20 sec for polymerase activation followed by 45 cycles of 95 ºC for 15 sec denaturation and 60 ºC for 60 sec annealing and extension. The Taqman probes were detected with the filter sets 470/514 nm for FAM, 533/572 nm for HEX, 577/620 nm for LC610, and 645/697 nm for Cy5.

Fold changes were calculated relative to the non-treated 0 h time point of each condition and biological replica. Data were normalized against RNase P internal control. qPCR efficiency values were calculated for each biological replica and included for fold change calculations. The average was calculated for three independent biological replicas to obtain the fold change of genomic and anti-genomic viral load, and transcriptional mRNA levels for each target gene. Data were analyzed with GraphPad Prism 6.0c.

#### Transcriptome sequencing

HeLa-MuV cells after ∼1 h of 0.5 mM As(III) were used for isolating total RNA according to manufacturer’s instructions using the RNeasy Micro Kit (Qiagen). Briefly, RNA integrity was checked using the RNA Nano 6000 Assay Kit of the Bioanalyzer 2100 system (Agilent Technologies), and concentration was measured with Qubit RNA Assay Kit in Qubit 2.0 Fluorometer (Life Technologies). Stranded mRNA-Seq libraries were prepared from 250 ng of total RNA using the NEBNext Ultra II Directional RNA Library Prep Kit for Illumina (New England Biolabs) implemented on the liquid handling robot Beckman i7. The protocol starts with the isolation of mRNA from total RNA by a polyA selection with oligo d(T) beads, followed by fragmentation and priming of mRNA with random primers to generate double-stranded cDNA fragments. Subsequently, adaptors were ligated to cDNA fragments, which were then amplified with 13 PCR cycles and purified with SPRIselect beads (Beckman Coulter). Fragmentation time purification steps were optimized to allow the selection of larger fragment sizes (>200 nt). Size distribution of the final library was assessed on Bioanalyzer with a DNA High Sensitivity kit (Agilent Technologies), and concentration was measured with Qubit DNA High Sensitivity kit in Qubit 2.0 Fluorometer. Final libraries were pooled in equimolar amounts and loaded at 2 pM solution on the Illumina sequencer NextSeq 500 MID output and sequenced unidirectionally, generating ∼150 million reads per run, each 155 bases long.

Sequencing reads were aligned using BWA-MEM (v0.7.17-r1188) with default parameters to the Enders strain of Mumps virus (GU980052).^76^ Duplicated reads were then filtered out using MarkDuplicates function in Picard tool (v2.9.0-1-gf5b9f50-SNAPSHOT). FreeBayes (v1.1.0-3-g961e5f3) was used to call single nucleotide variations compared to the Enders strain sequence. Finally, the variants were filtered based on their qualities (> 20).

#### RNA Fluorescence in situ hybridization (FISH) and immuno-RNA FISH

Probe sets targeting genomic RNA of MuV-N or MuV-L regions were designed, labeled and purified according to Gaspar et al.^101^ with the unlabeled DNA oligonucleotides synthesized from Sigma-Aldrich (40 oligonucleotides for MuV-N and 80 oligonucleotides for MuV-L) as listed in Table S1. The concentrations of Atto488-labeled probe sets targeting MuV-N or MuV-L were measured to be 14.1 μM or 19.4 μM, respectively. One day before the assay, HeLa-MuV cells were seeded on 8-well slides (Ibidi, μ-Slide), with 20,000 cells per well. The next day, cells were stressed with 1 mM As(V) or 30 μM As(III) for 6 h, or not treated as control. Then cells of both stress and control conditions were briefly rinsed 2-3 times with warm PBS, before being fixed with 4% PFA for 10-15 min. After rinsing again with PBS for 2-3 times, cells were permeabilized the with 70% ethanol at 4 ºC for 2 h. Next, the ethanol was aspirated and cells incubated with Stellaris RNA FISH Wash Buffer A (with 10% deionized formamide; Biosearch Technologies) at room temperature for 2-5 min. In a humidified chamber, the Wash Buffer A was aspirated followed by incubation in Stellaris RNA FISH Hybridization Buffer (with 10% deionized formamide; Biosearch Technologies) containing probe sets targeting genomic regions of MuV-N or MuV-L (2 nM per oligonucleotide as the working concentration). The entire chamber was sealed and incubated in the dark at 37 ºC for ∼15 h. The Hybridization Buffer was then aspirated and the sample incubated again with Wash Buffer A in the dark at 37 ºC for 30 min, then with Stellaris RNA FISH Wash Buffer B (Biosearch Technologies) in the dark at room temperature for 2-5 min. Then immunostaining was performed with primary antibody against the MuV-N protein in the same way as above, except changing the secondary antibody to goat anti-mouse IgG Alexa Fluor plus 647 (1:1,000; highly cross-adsorbed; Invitrogen, A-32728). Imaging was performed on the Olympus FV3000 confocal microscope with a 60 × 1.3 NA silicone oil immersion objective and GaAsP PMT detectors. Central slice images were acquired for colocalization analysis of the FISH channel with the anti-N immunostaining channel.

The sequential staining of RNA first and then IF resulted in dim signal of the FISH channel, while IF before RNA FISH resulted in almost no signal of the FISH channel, likely due to competing binding of the antibody and FISH probes which both target the MuV nucleocapsids. Thus, for quantification of the RNA FISH signal at stress condition versus control, RNA FISH only experiments were carried out, in the same way as for immuno-RNA FISH until the Wash Buffer B incubation step and nuclei were stained with mounting medium with DAPI. The protocol was optimized to gain better signal of the FISH channel by increasing the concentration of the deionized formamide in the Hybridization Buffer and the Wash Buffer A to 15% from 10%. Images were taken on the LSM780 as Z-stack with a Z-step size of 0.22 μm (131.8 nm per pixel). Segmentation and quantification of FISH channel signal (viral factories) were performed in Imaris, similarly as above for IF using the Surface module. Surfaces of FISH signal structures were generated by absolute intensity thresholding with a fixed value and surfaces of nuclei by background subtraction auto-thresholding of the DAPI staining signal, separately. Distances between viral factories and nuclei were calculated for each image stack with MATLAB scripts to assign viral factories to individual cells. The sum intensity of FISH channel signal per cell was then used for generating plots with RStudio scripts.

#### Western blot

HeLa-MuV cells were seeded ∼24 h prior to the start of stress treatment in T-25 flasks for each sample. Stress treatment was performed by adding 100 μl of 1.5 mM sodium arsenite solution into each T-25 flask (final concentration of 30 μM in 5 ml culture medium each) and gently mixing it with culture medium. Samples were collected at four time points (0 h, 6 h, 12 h, and 24 h) for both stressed and control samples. First, the culture medium of each was separately collected for subsequent enrichment of the released N protein. Then, cell pellet samples were collected *via* direct lysis by adding 1% SDS dissolved in PBS, with 1.5 mM MgCl_2_, EDTA-free protease inhibitor cocktail (cOmplete, Roche) and 1 mM PMSF. 0.3 μl of Benzonase (250 U/μl, Millipore) was added to each flask and the flasks were shaken on a shaking platform (Eppendorf Thermomixer Compact) at 750 RPM for 15-20 min until the jelly substance disappeared. The cell lysate was then recovered from the flasks, adjusted to the same volume, heated at 95 ºC for 5 min and frozen for further use. Processing of the culture medium containing the released viral proteins was started by centrifugation at 2,200 *g*, 4 ºC for 15 min to remove cell debris. Then, the supernatant was mixed with 4× concentration solution (40% PEG 6,000, 100 mM HEPES, pH 7.5, and 2 M NaCl, with EDTA-free protease inhibitor cocktail and 1 mM PMSF) thoroughly and left at 4 ºC. After ∼24 h of precipitation, the samples were centrifuged at 4,300 *g*, 4 ºC for 30 min. After careful removal of the supernatant, a white disk was seen at each tube bottom, and recovered with the same lysis buffer as for cell pellets. Volumes of these samples containing the released viral proteins were also adjusted to the same for all samples, heated at 95 ºC for 5 min and frozen for further use.

Total protein concentration of each cell lysate sample was determined by Bradford assay with Protein Assay Dye (Bio-Rad) on a UV/Visible spectrophotometer (Ultrospec 2100 pro, Amersham Biosciences). For western blot analysis, volume corresponding to ∼20 μg total protein was taken for each cell lysate sample and supplemented to the same volume for all samples before the LDS sample buffer with reducing agent (Invitrogen) was added. The samples containing the released viral proteins were also volume adjusted according to the total protein concentration in their respective cell lysate sample so that the comparison will be performed with the same total intracellular protein level among all time points, for both the stressed and control samples. Proteins were separated using NuPAGE 4-12% precast Bis-Tris protein gels in MOPS SDS running buffer (Invitrogen), transferred onto Immobilon-P PVDF membranes (Millipore), and analyzed with mouse monoclonal antibodies against MuV-N (1:1,000; Abcam) and glyceraldehyde-3-phosphate dehydrogenase (GAPDH, 1:4,000; Santa Cruz) as loading control. Goat anti-mouse IgG (H/L):HRP (1:1,000; multi species absorbed; Bio-Rad) and ECL western blot detection reagents (Cytiva) were then used for detection with a ChemiDoc Imager (Bio-Rad). Intensity quantification was done in Image Lab (v6.1; Bio-Rad). To calculate the intracellular levels of MuV-N over time (fold change), the intensity of N in cell lysate samples was normalized to the intensity of the respective loading control (GAPDH) and shown as relative to 0 h. To analyze the released levels of N over time (fold change), the intensity of MuV-N in samples containing the released viral proteins was divided by the intensity of MuV-N in the respective cell lysate sample and shown as relative to 0 h. Data were analyzed with GraphPad Prism 6.0c.

#### Mass spectrometry (MS) cell lysate preparation

##### Whole cell lysate preparation

Cells were stressed as described above. At the indicated time points, cells were detached by trypsinization, washed and pelleted twice with PBS. For total proteome analysis, 0.5 million cells per condition were lysed with 100 μl of PBS containing 1.5 mM MgCl_2_, 1% SDS and 0.25 U/μl Benzonase and incubated at room temperature for 15 min. The protein concentration in cell lysates were determined using a BCA assay. Cell lysate volumes corresponding to 5 μg total protein from different conditions were utilized for MS sample preparation.

##### Solubility proteome profiling (SPP) lysate preparation

One cell pellet containing 1 million cells was frozen per time point following cellular stress (as described above). The cell pellets were thawed on ice and lysed (3 freeze thaw cycles) after re-suspending each one in 100 μl of PBS containing protease inhibitors (Roche), 0.8 % NP40, 1.5 mM MgCl_2_ and phosphatase inhibitor (Roche). The lysate was split into two 50 μl aliquots. One aliquot was spun at 100,000 *g*, 20 min at 4˚C and the supernatant was retrieved. This represented the “soluble-NP40” fraction of proteins. The second aliquot was further solubilized with SDS (final concentration 1%). This represented the “total-SDS” proteome. Both aliquots were incubated with Benzonase (final concentration 0.25 U/μl). The protein concentration of total proteome fractions was determined with BCA assay. Volumes of total lysate corresponding to 5 μg protein from different conditions were calculated. Equal volume of the soluble-NP40 samples to its corresponding total lysate was utilized. Both soluble and total lysate of each condition over the time course was multiplexed as a single MS experiment.

#### MS sample preparation

MS sample preparation and measurements were performed as described.^42^^,^^102^ Protein digestion was performed using a modified SP3 protocol.^102^^,^^103^ 5 μg of proteins (per condition) were diluted to a final volume of 20 μl with 0.5% SDS and mixed with a paramagnetic bead slurry (10 μg beads (Sera-Mag Speed beads, Thermo Fischer Scientific) in 40 μl ethanol). The mixture was incubated at room temperature with shaking for 15 min. The beads now bound to the proteins were washed 4 times with 70% ethanol. Proteins on beads were reduced, alkylated and digested using 0.2 μg trypsin, 0.2 μg LysC, 1.7 mM TCEP and 5 mM chloroacetamide in 100 mM HEPES, pH 8. Following an overnight incubation, the peptides were eluted from the beads, dried under vacuum, reconstituted in 10 μl of water and labelled with TMT-10 or TMT-16 plex reagents and dissolved in acetonitrile at 1:15 (peptide:TMT weight ratio) for one hour at room temperature. The labelling reaction was quenched with 4 μl of 5% hydroxylamine and the conditions belonging to a single MS experiment were pooled together. The pooled sample was desalted with solid-phase extraction after acidification with 0.1% formic acid. The samples were loaded on a Waters OASIS HLB μelution plate (30 μm), washed twice with 0.05% formic acid and finally eluted in 100 μl of 80% acetonitrile containing 0.05% formic acid. The desalted peptides were dried under vacuum and reconstituted in 20 mM ammonium formate. The samples were fractionated using C18-based reversed-phase chromatography running at high pH. Mobile phases constituted of 20 mM Ammonium formate pH 10 (buffer A) and acetonitrile (buffer B). This system was run at 0.1 ml/min on the following gradient: 0% B for 0 – 2 min, linear increase 0 – 35% B in 2 – 60 min, 35 – 85% B in 60 – 62 min, maintain at 85% B until 68 min, linear decrease to 0% in 68 – 70 min and finally equilibrated the system at 0% B until 85 min. Fractions (0.2 ml) were collected between 2 – 70 min and every 12^th^ fraction was pooled together and vacuum dried.

#### LC-MS-MS measurement

Samples were re-suspended in 0.05% formic acid and analyzed on Q Exactive Plus or Orbitrap Fusion Lumos mass spectrometers (Thermo Fischer Scientific) connected to UltiMate 3000 RSLC nano system (Thermo Fisher Scientific) equipped with a trapping cartridge (Precolumn; C18 PepMap 100, 5 μm, 300 μm i.d. × 5 mm, 100 Å) and an analytical column (Waters nanoEase HSS C18 T3, 75 μm × 25 cm, 1.8 μm, 100 Å) for chromatographic separation. Mobile phase constituted of 0.1% formic acid in LC-MS grade water (Buffer A) and 0.1% formic acid in LC-MS grade acetonitrile (Buffer B). The peptides were loaded on the trap column (30 μl/min of 0.05% trifluoroacetic acid in LC-MS grade water for 3 min) and eluted using a gradient from 2% to 30% Buffer B over 2 h at 0.3 μl/min (followed by an increase to 40% B, and a final wash to 80% B for 2 min before re-equilibration to initial conditions). The outlet of the LC- system was directly fed for MS analysis using a Nanospray-Flex ion source and a Pico-Tip Emitter 360 μm OD × 20 μm ID; 10 μm tip (New Objective). The mass spectrometer was operated in positive ion mode. The spray voltage and capillary temperature was set to 2.3 kV and 275°C respectively. Full-scan MS spectra with a mass range of 375–1,200 m/z were acquired in profile mode using a resolution of 70,000 (maximum fill time of 250 msec or a maximum of 3e^6^ ions (automatic gain control, AGC)). Fragmentation was triggered for the top 10 peaks with charge 2–4 on the MS scan (data-dependent acquisition) with a 30-sec dynamic exclusion window (normalized collision energy was 30), and MS/MS spectra were acquired in profile mode with a resolution of 35,000 (maximum fill time of 120 msec or an AGC target of 2e^5^ ions).

#### MS protein identification and quantification

The MS data was processed as previously described.^42^ Briefly, the raw MS data was processed with isobarQuant, and identification of peptides and proteins was performed with Mascot 2.4 (Matrix Science) against a database containing *Homo sapien* Uniprot FASTA (proteome ID: UP000005640, downloaded on 14 May 2016)^94^ and in-house sequenced and assembled encoding sequences of Mumps Enders strain (single nucleotide variations see Key Resources Table) along with known contaminants and the reverse protein sequences (Human-MuV DB, search parameters: trypsin; missed cleavages 3; peptide tolerance 10 ppm; MS/MS tolerance 0.02 Da; fixed modifications included carbamidomethyl on cysteines and TMT-10 or TMT-16 plex on lysine; variable modifications included acetylation of protein N-terminus, methionine oxidation and TMT-10 or -16 plex on peptide N-termini). The whole cell proteome datasets were also searched with phosphorylation on S|T|Y as a variable modification.

#### MS Data analysis

All MS data analysis was performed using R (v.3.6.1).

##### Whole cell proteome data analysis

The distributions of signal sum intensities from all TMT channels was normalized with vsn^95^ to correct for slight differences in protein amounts. Differential analysis was performed on log_2_ transformed signal sums of different stress time points and control condition using limma.^96^ Proteins with |log_2_(fold change) | > 0.5 and adjusted p-value (Benjamini Hochberg) < 0.01 were considered significantly changed.

##### Solubility proteome profiling data analysis

Data normalization for solubility proteome profiling experiments were performed based on a subset of proteins that are predominantly soluble. The NP40/SDS ratio of proteins was calculated using raw signal sum intensities. Proteins with NP40/SDS ratio between 0.8 and 1.2 represented the soluble subset. This subset was utilized for calculating the calibration and transformation parameters for vsn.^95^ These parameters were then applied to all proteins identified to correct for technical variations. The NP40/SDS ratio was calculated using normalized signal sum intensities and log_2_ transformed for performing differential analysis using limma.^96^ Proteins with |log_2_(fold change) | > 0.5 and adjusted p-value (Benjamini-Hochberg) < 0.1 were considered significantly changed.

##### Gene ontology (GO) overrepresentation analysis

Differentially expressed human proteins from 30 μM arsenite (As(III)) treatment dataset were used for GO term “Biological processes” overrepresentation analysis using clusterProfiler (R Bioconductor).^97^ All identified proteins in this dataset were used as the background. Standard settings were used for representing enriched GO terms (p-value cutoff: 0.05, Benjamini-Hochberg procedure for multiple testing adjustment and q-value cutoff of 0.2).

#### Structural modeling of P-L complex

The P-L complex structures of parainfluenza virus 5 (PIV5) and MuV at the P-L interface were modeled using AlphaFold2 (AF2)^48^ with all parameters set to default. Modeling was started with the PIV5 P-L complex. To be noted, although modeling with one molecule of full-length L and four molecules of P was initially attempted, only modeling with domains of P and L involved in their interaction in agreement with the published model of PIV5 P-L complex provided results that can fit well into the published EM map (EMD: 21095). In the end, sequences used as input are the following: for PIV5, one molecule of L (amino acid residues [aa.] 1-912), one molecule of P (aa. 199-392) and three molecules of P (aa. 199-274); similarly, for MuV, one molecule of L (aa. 1-913), one molecule of P (aa. 213-391) and three molecules of P (aa. 213-273). The predicted PIV5 P-L complex model was first fitted into the published EM map as a rigid body and regions not built in the reported structure fitted well to the unassigned density of the EM map at secondary structure level. Then, the model-map fitting was optimized with Isolde plugin in UCSF ChimeraX^90^ (v1.2.5) for the flexible linker regions. The quality of the predicted model was assessed by the predicted local distance difference test (pLDDT) and predicted aligned error (PAE) scores generated by AF2. Although regions with no clear secondary structures (α-helix or β-strand) had relatively low pLDDT score, the newly-fitted neighboring regions with secondary structures were used to validate the location and orientation of linker regions. The AF2 generated model of the MuV P-L complex was next matched to the PIV5 P-L model in Chimera^89^ and the region corresponding to the detected phosphorylation sites in P of MuV was mapped to the P-L interface. Surface charge representations of P at the interface with L in P_WT versus a mimic of the phosphorylated P (S mutated to D and T mutated to E; called P_DDED) were generated with models predicted separately for P_WT and P_DDED.

#### Nucleocapsids isolation and negative staining electron microscopy

HeLa-MuV cells were seeded on 20-cm dishes one day prior to stress treatment. After replacing the culture medium with that containing 0.5 mM As(III) and incubating the cells for ∼1 h, cells were scraped off dishes with 2 ml medium per dish, pelleted at 850 *g* for 3 min and frozen and stored at -80 ºC. On the day of the experiment, the cell pellets were thawed on ice and resuspend with 250 μl of lysis buffer (50 mM Tris, pH 7.5, 100 mM KAc, 2 mM Mg(Ac)_2_, 0.5 mM DTT, 0.5% IGEPAL CA-630, EDTA-free protease inhibitor cocktail, 1 U/μl Ribonuclease inhibitor Sigma) per vial of pellet. The following operations were performed on ice unless specified. Cells were lysed with a syringe with a 27G ¾ needle for 5 passages. The lysate was centrifuged at 500 *g*, 4 °C, for 5 min to pellet cell debris. Next, the supernatant was centrifuged at 13,000 *g*, 4 °C, for 20 min. The pellet was sufficient for enriching nucleocapsids without significant cellular contaminants. Pellets (resuspended in the lysis buffer without IGEPAL CA-630) and supernatants of each centrifugation step were used for negative staining electron microscopy (EM) experiments. Nucleocapsids in the 13,000 *g* pellet resuspension were additionally supplied with heparin solution at 50 μg/ml for 2 h. For generating data presented in Figure S5B, nucleocapsid were isolated in the same way except that fresh medium was used for the non-stress control, or that containing 1 mM As(V) for the 6 h stress treatment; no heparin was added.

Negative staining was done with 1% uranyl acetate solution. For each sample, 2.5 μl of sample solution (diluted by 1:4 to 1:100 depending on the concentration) was deposited on an EM copper grid (400-mesh, Plano G2400C, coated with 6 nm thick carbon produced with a Leica EM ACE600 sputter coater), incubated for 30–60 sec and manually blotted with filter paper. 2.5 μl of uranyl solution was then immediately applied to the grid and blotted away, and this step was repeated for three times. Grids were left to dry and then imaged with a Tecnai T12 EM operated at 120 kV (Thermo Fisher Scientific) at 13,000× magnification for screening and 49,000× magnification for image acquisition.

#### Sucrose gradient fractionation and MS profiling

Sucrose gradient fractionation protocol was optimized in three experiments with samples from 1 h of 0.5 mM As(III) treatment or 6 h of 1 mM As(V) treatment. In the initial experiment, the cell lysate after removal of cell debris at 850 *g* was first pelleted with an Optima MAX-XP Ultracentrifuge (with TLA-100 rotor, Beckman Coulter) at 35,000 rpm (47,265 *g*) 4 °C, for 40 min in order to collect most nucleocapsids. The pellet was then resuspended in lysis buffer without IGEPAL CA-630, and loaded on a 5 ml 10-65% (w/v) sucrose gradient prepared with a BioComp Gradient Master instrument. Ultracentrifugation was carried out at 35,000 rpm (116,140 *g*), 4 °C for 2 h in an Optima L-100XP ultracentrifuge (with SW 55 Ti rotor, Beckman Coulter). Fractionation was done manually by collecting every 200 μl volume from the top to the bottom of each centrifuge tube. Every other fraction was imaged by negative stain EM. Based on the images, the pellet at 35,000 rpm contained too much cellular material. Thus, the experiment was optimized for two more times, using the 13000 *g* pellet as the starting material for a sucrose gradient of 20-70%, or 20-60% (the latter used for MS profiling analysis). Sucrose in fractions were removed by dialysis with the same lysis buffer without IGEPAL CA-630, in order to reduce background for negative staining EM. Fractions around the one showing the most abundant nucleocapsids in negative staining EM were subjected to mass spectrometry analysis as described above.

For data shown in Figures 4D–4E and S3G–S3H, in order to estimate the relative protein amounts in the arsenate (As(V)) treated and control samples prior to sucrose fractionation, we analyzed aliquots of the unfractionated samples from both conditions. The ratio of signal sum intensities of proteins between the unfractionated arsenate treated and control samples was calculated to correct for variations in input. The relative fold-changes of proteins in individual fractions of sucrose gradient (in control and arsenate treated sample) was calculated with reference to fraction 10. The apex of the peak for protein N, L, P and M was detected at fraction 14 in arsenate treatment fractionation and fraction 15 in control fractionation. The ratio of above-mentioned relative fold change in arsenate (fraction 14) and control (fraction 15) was calculated for N, L and P. This ratio was further corrected for variation in the input used for fractionation. The corrected ratio represented the amount of N, L and P proteins at the apex of the sucrose gradient fractionation in arsenate treated compared to control cells.

#### Immunoprecipitation-MS

HeLa-MuV cells were transfected with EGFP- P plasmids (WT, or P_deficient mutant) as described above. At 24 h after transfection, cells were collected by trypsinization, counted with a TC20 automated cell counter (Bio-Rad), frozen and stored at -80 °C until use. Cells transfected with P_WT for comparison of stress versus control were either treated with 1 mM As(V) for 6 h or not treatment before collection. Cells transfected with P_WT or P_deficient for comparison of the effect of mutations were not stressed before collection. On the day of immunoprecipitation (IP), three million cells in three replicates per condition were lysed in the same manner as for nucleocapsid isolation above. The lysate was centrifuged at 850 *g*, 4 °C, for 5 min and the supernatant was used as input for EGFP-P pulldown with GFP-Trap magnetic agarose (ChromoTek) according to the standard protocol. Buffer containing 50 mM Tris, pH 7.5, 100 mM KAc, 2 mM Mg(Ac)_2_ was used for washing and LDS sample buffer (Invitrogen; with DTT freshly added to 0.1 M at the working concentration) was used for elution of proteins from beads with 10 min incubation at 95 °C. Specifically, for IP of P_WT at stress versus control, the supernatant of 850 *g* centrifugation was subjected to 13,000 *g* centrifugation as for isolation of nucleocapsids and the 13,000 *g* pellet was used as input for IP. For IP of P_WT versus P_deficient, the supernatant of 850 g centrifugation was used instead to increase input protein amount and better capture potential changes of P-L association in relation to mutations of P. Both IP input and eluate samples were stored at -20 °C until prepared and labelled for MS analysis.

Sample preparation, TMT-labelling for multiplexing and MS analysis was performed as described above with minor variations as following: (a) multiplexed IP input samples were fractionated using high-pH reversed phase LC (as described previously) and collected into 6-fractions for MS analysis, (b) multiplexed IP eluate samples were desalted using OASIS HLB μelution plate (30 μm) (as described previously) and directly analyzed on MS without prefractionation owing to low proteome complexity. *Data search:* MS data was searched by including the sequence of transfected EGFP-P protein into the Human-MuV DB using similar search parameters as described above. Where indicated, the data was re-searched with phosphorylation on S|T|Y as a variable modification to infer on phosphorylated version of P. *Data analysis*: The signal sum intensities of protein identified from all conditions (for all replicates) were normalized using vsn.^95^ The normalized protein intensities (stress versus control cells or P_WT versus P_deficient) was corrected for any variations in the input by calculating the ratio between eluate and input per protein per condition. Next, both stress and control conditions were normalized for the amount of EGFP-P pulled down in each condition (to correct for the IP efficiency; see key resources table). Differential analysis to assess significantly changing protein interaction between conditions was performed with limma.^96^

#### Plunge freezing

The isolated nucleocapsids were prepared from the lysate of HeLa-MuV cells after 1 h of 0.5 mM As(III) stress as above (13,000 *g* pellet, 50 μg/ml heparin treated). Plunge freezing was done with a Vitrobot Mark IV (Thermo Fisher Scientific) set to 22 °C, 100% humidity. Glow-discharged holey Quantifoil grids with an additional layer of continuous carbon (R2/1 + 2 nm C, Cu 200 mesh grid, Quantifoil Micro Tools) were used. 3 μl of nucleocapsids suspension was mixed with 1.5 μl of Protein-A, 10-nm colloidal gold (in the same buffer as nucleocapsids; Electron Microscopy Sciences) and deposited onto grids. Grids were blotted from both sides with blot force 0 for 2 sec and drained for 2 sec before being plunged into liquid ethane at liquid nitrogen temperature. The frozen grids were stored in sealed boxes in liquid nitrogen until imaging.

#### Sample preparation and vitrification for cellular cryo-ET

For plunge freezing of HeLa-MuV cells under different stress conditions as described above, either a Vitrobot Mark 4 or Leica EM GP (Leica Microsystems) were used. Gold Quantifoil grids (R1/4, Au 200 mesh grid, SiO_2_, Quantifoil Micro Tools) were glow-discharged and UV irradiated for 30 min for sterilization before being immersed in cell culture medium in 35-mm Ibidi μ-Dish. Next, HeLa-MuV cells were seeded in such dishes each containing 5-6 grids and cultured in an incubator overnight at 37 °C and 5% CO_2_. Cells cultivated on grids were plunge-frozen in liquid ethane/propane mixture at close to liquid nitrogen temperature. The blotting conditions for the Vitrobot were set to 37 °C, 90% humidity, blot force 10, 10 sec blot time and 2 sec drain time and grids were blotted from the reverse side with the aid of a Teflon sheet from the front side. The blotting conditions for the Leica EM GP were set to 37 °C, 90% humidity, blot volume 3 μl, 2-3 sec blot time and grids were also blotted from the reverse side. For grids that were used in subsequent correlative imaging, 2 μl of 1-μm crimson beads (FluoSpheres carboxylate-modified microspheres, 625/645, Thermo Fisher Scientific) diluted 1:40 from original stock were added to the grid surface from one side before blotting. Grids were stored in liquid nitrogen until usage.

#### Cryo-fluorescence light microscopy

Frozen grids were fixed into custom-made AutoGrid specimen cartridges modified for FIB preparation under shallow angles^104^ and imaged by a prototype Leica cryo-fluorescence light microscope (FLM) based on a Leica TCS SP8 CFS equipped with a cryo-stage operated at liquid nitrogen temperature.^105^ The microscope includes a widefield light path and a confocal path with independent light sources and is equipped with a 50× 0.9 NA cryo-objective. The grid overview was first acquired using the widefield path. Intact grid squares with signal of interest were then imaged with the confocal path by exciting with a 552-nm laser and simultaneously detecting at 569-633 nm and 695-700 nm with two HyD detectors. After imaging, grids were stored in liquid nitrogen for the next step.

#### Cryo-focused ion beam milling

Plunge-frozen grids fixed into custom-made AutoGrids were mounted into a 45° pre-tilt shuttle and transferred into an Aquilos cryo-focused ion beam/scanning electron microscope (FIB/SEM dual-beam microscope, Thermo Fisher Scientific). During FIB operation, samples were kept at constant liquid nitrogen temperature. To improve sample conductivity and reduce curtaining artifacts during FIB milling, the samples were first sputter-coated with platinum and then coated with organometallic platinum using the gas injection system.^106^ Lamellae were prepared using Gallium ion beam at 30 kV and stage tilt angles of 15°-20°. The MAPS software installed on the Aquilos microscope was used to refine eucentricity and record coordinates to prepare lamellae. Lamella preparation was conducted in a stepwise manner: rough milling with currents of 1 nA, gradually reduced to lower currents, down to 50 pA for the final polishing step.^25^ Progress of the milling process was monitored using the SEM operated at 10 kV and 50 pA. For improving conductivity of the final lamella, we sputter-coated the grid again with platinum. The thickness of the platinum layer and the lamellae were determined from the tomographic reconstructions to be approximately 5 nm and 120-250 nm, respectively.

For lamella preparation following a 3D correlative workflow, the MAPS software provides 3-point correlation (based on features of grid squares) between the cryo-FLM and cryo-SEM images for 2D navigation in order to find squares for which the cryo-FLM data were acquired. About 10 microbeads were picked in the squares of interest and correlated between the cryo-FLM stacks (3D Gaussian plot to optimize geometry center) and the SEM images, as well as the FIB images (2D Gaussian plot to optimize geometry center) using 3DCT software (v2.2.2).^80^ After choosing the signal of interest in the confocal stacks, 3DCT provided the position as the center to place the parallel rectangular milling patterns above and below the center along the Y axis on FIB images, in order to retain the signal (region of interest) in the lamella. Milling was then performed as described above. After lamella generation, 3DCT was again used to correlate the cryo-FLM data with the SEM and FIB images to register the signal of interest onto the lamella.

#### Cryo-electron tomography data collection

Cryo-electron tomography (ET) data of isolated nucleocapsids were collected on a Titan Krios microscope operated at 300 kV (Thermo Fisher Scientific) equipped with a field-emission gun, a Quantum post-column energy filter (Gatan) and a K2 direct detector camera (Gatan). Data were recorded in dose-fractionation mode using acquisition procedures in SerialEM software^77^ (v3.7.2). Prior to the acquisition of tilt-series, montages of the grid squares were acquired at 3.106 nm/pixel to identify filamentous structures which were then confirmed as nucleocapsids at higher magnification. Tilt-series were collected for nucleocapsids using a dose symmetric scheme^78^ in nano-probe mode, calibrated pixel size at the specimen level of 1.6938 Å, defocus range 2.5 to 3.5 μm, tilt increment 3° with constant dose of 2.5 e^−^/Å^2^ for all tilts, tilt range -60° to 60° starting from 0°, a total dose of ∼102.5 e^−^/Å^2^. In total, twenty tomograms were acquired for the isolated nucleocapsids.

Cryo-ET data of FIB lamellae from cells at ∼1 h of 0.5 mM As(III) stress for subtomogram averaging purpose were collected with the same Titan Krios microscope, except a pixel size of 2.284 nm used for overviews and 3.3702 Å/pixel for tilt series, defocus of 3.25 to 3.5 μm, increment 2° with constant dose of 2.5 e^−^/Å^2^ for all tilts, tilt range -60° to 54° starting from -12° (lamella pre-tilt angle), a total dose of ∼145.0 e^−^/Å^2^. The AutoGrids with lamellae were carefully loaded with the lamella orientation (cut-out direction on custom-made AutoGrids) perpendicular to the tilt axis of the microscope for tilt series acquisition. For lamellae that were prepared following a 3D correlative approach, TEM overviews of lamella were overlaid with their SEM image that were already superimposed with the respective confocal oblique slice images (computed with MATLAB scripts), using the SerialEM registration points strategy. Two tomograms containing nucleocapsids were used for subsequent analyses.

Cryo-ET data of FIB lamellae from cells at ∼2 h of 0.5 mM As(III), ∼6 h of 30 μM As(III), or ∼6 h of 1 mM As(V) stress conditions were collected on a different Titan Krios microscope equipped with a K3 direct detector camera (Gatan). Differences in settings are pixel size for lamella overview of 2.804 nm/pixel and for tilt series of 1.631 Å/pixel, defocus of 1.75 to 3.25 μm, increment 2° with constant dose of 2.6 e^−^/Å^2^ for all tilts, tilt range -60° to 60° (starting angle depending on pre-tilt angles of lamellae), a total dose of ∼158.6 e^−^/Å^2^. In total, three tomograms containing nucleocapsids for ∼2 h of 0.5 mM As(III), one tomogram for ∼6 h of 30 μM As(III), and five tomograms for ∼6 h of 1 mM As(V) were used for subsequent analyses.

In addition to tomograms described above, which were used for subtomogram averaging, cryo-ET data of nucleocapsids *in situ* at control and ∼1 h of 0.5 mM As(III) stress were also collected with a Volta phase plate^107^ (VPP, Thermo Fisher Scientific) for the purpose of better visualization. The operation of the VPP were carried out as described previously, applying a beam tilt of 10 mrad for autofocusing.^108^ Lamella maps were acquired at 2.284 nm/pixel and tilt series acquired at 3.3702 Å, defocus of 2 to 3.75 μm, increment 2° with constant dose of 2.20 e^−^/Å^2^ for all tilts, tilt range -64° to 50° starting from -8°, and a total dose of ∼127.6 e^−^/Å^2^. In total, two tomograms containing nucleocapsids for control condition and one tomogram for ∼1 h of 0.5 mM As(III) stress were used here for visualization.

#### Tomogram reconstruction

For processing of cryo-ET data of the isolated nucleocapsids, frames of the projection movies were imported to Warp software^81^ (v1.0.9) for gain reference and beam-induced motion correction, as well as contrast transfer function (CTF) and astigmatism estimation. Prior to tomogram reconstruction in Warp, per-tilt averaged images were imported to IMOD^79^ (v 4.9.4) for tilt series alignment. Alignment of tilt-series images was performed with patch-tracking due to insufficient gold fiducials for tracking. Final alignment was done using the linear interpolation option in IMOD. Aligned images were initially 4 times binned to a pixel size of 6.7752 Å. Reconstruction was done in Warp at the same pixel size by importing the transformation files from IMOD. Deconvolved tomograms at the same binning were also reconstructed in Warp for segmentation purpose.

For processing of cryo-ET data of nucleocapsids *in situ* at ∼1 h of 0.5 mM As(III) stress, which were used for subtomogram averaging, the same procedures were followed except that initial tomograms were reconstructed by 4 times binning to a pixel size of 13.4808 Å. Neural network-based denoising was done in Warp at the same binning to generate tomograms for segmentation purpose.

For processing of cryo-ET data of nucleocapsids *in situ* at ∼6 h of 30 μM As(III) stress or ∼6 h of 1 mM As(V) stress, which were used for subtomogram averaging, the same procedures were followed except that gain reference correction and motion correction were done in the SerialEM plugin, prior to Warp processing which started from per-tilt averaged images. Initial tomograms were reconstructed by 8 times binning to a pixel size of 13.048 Å. Neural network-based denoising was done in Warp at the same binning to generate tomograms for segmentation purpose.

For processing of cryo-ET data of nucleocapsids *in situ* at control and ∼1 h of 0.5 mM As(III) stress for visualization purpose, frames of the projection movies were gain reference and motion corrected in the SerialEM plugin. Tilt-series alignment and tomographic reconstructions were done in IMOD. Alignment of tilt-series images was performed with patch-tracking. Final alignment of the tilt-series images was performed using the linear interpolation option in IMOD without contrast transfer function correction. Aligned images were 4 times binned to a pixel size of 13.4808 Å. For tomographic reconstruction by back-projection, the radial filter options were left at their default values (cut off, 0.35; fall off, 0.05).

#### Filament tracing, volume fraction and persistence length analysis

Segmentation of *in situ* nucleocapsids was performed with the Fiber Tracing module in Amira software (v6.7 and v2019.4; Thermo Fisher Scientific), using the deconvolved or denoised tomograms. The approximate outer and inner diameters, and the helical pitch of nucleocapsids were measured from raw tomograms by averaging the values of multiple measurements, and used as input for the tracing parameters and subsequent sampling. The coordinates of the traced filaments were resampled with a MATLAB script to obtain equidistant points along the filament every 5.4 nm (4 pixels in datasets of 13.4808 Å/pixel). The approximate outer diameter of nucleocapsids as 21.30 nm was used for calculating the volumes of nucleocapsids per tomogram with the total lengths of nucleocapsid filaments calculated from the tracing. Volume of regions per tomogram that contain nucleocapsids was obtained by manually segmenting areas where filaments were seen in Amira. The volume faction of nucleocapsids was then derived by dividing the volume of nucleocapsids to the total volume of regions containing nucleocapsids for individual tomograms. Tomograms with too few nucleocapsids (very small fractions of viral factories captured) were not used for volume fraction analysis.

Quantitative assessment of the morphology of nucleocapsid filaments was obtained by measurement of the persistence length (L_p_), the distance for which a filament’s direction persists before changing its course.^109^ To determine its magnitude, we measured the difference between an angle θ_l_ between the tangents at points of distance l along the curved filament with respect to a fixed point on the filament l_0_ with angle θ_0_. Doing so for an ensemble of filaments in the tomographic volume, the correlation is determined by <cos(θ_0_- θ_l_) >= e^–l/Lp^. Mean length of filaments for each tomogram was determined to define the length range used for fitting of the slope. Persistence lengths of microtubules, actin, and nuclear lamina were determined *in situ* to be 12.07×10^3^, 2.79×10^3^, and 555.57 nm respectively in a previous report.^25^ Nucleocapsids in our data are very flexible based on the persistence length comparison with these cytoskeletal filaments. Tomograms with too few nucleocapsids (very small fractions of viral factories captured) were not used for persistence length analysis.

#### Tomogram segmentation

For visualization of ribosomes and membranes in the tomogram shown in Figure 5B, in-house developed 3D convolutional neural networks for ribosomes localization and membranes segmentation were pretrained with large datasets and used here for prediction.^98^ The prediction output was inspected and cleaned manually with Amira and the final figure was generated with ChimeraX.

#### Subtomogram averaging of isolated nucleocapsids

For subtomogram averaging of the isolated nucleocapsids, nucleocapsids were first traced in Dynamo package^82^ (v1.1.401) with the 4 times binned tomograms. In the Dynamo model generation interface, extremal points of relatively straight segments along each nucleocapsid were manually selected to define the central line “backbone”. Bent regions were discarded. Directionality of individual nucleocapsids were visually inspected and considered when selecting extremal points. Successive orthogonal sections along the nucleocapsid backbone were then generated such that central points of these sections were manually adjusted to define series of ordered points composing the refined backbone. After that, the Dynamo filament model with torsion type was used for generation of subtomograms (called “subunits” in Dynamo). The torsion effect in this model refers to the fact that the x direction of two subsequent subtomograms can be chosen to vary by a fixed angle. The two parameters subunits_dz and subunits_dphi were set as 8 pixels (5.4 nm) and 30°, considering the helical pitch and nucleoprotein subunits per turn roughly measured from tomograms, as well as for reducing the effect of missing wedge in subtomogram averaging.^110^ Subtomograms (4 times binned, box size of 64^3^ pixels) were cropped using Dynamo with the model-defined positions and initial Euler angles were generated relative to filament axes with extra in-plane rotations derived from torsion angles (Figure S5F).

Reference-free subtomogram averaging/refinement was performed using Dynamo, adapted TOM^83^ and AV3^84^ (TOM/AV3) software toolboxes and derived MATLAB scripts, RELION^85^ (v3.0 and v3.1), Warp and M^24^ (v1.0.9). Initial reference was generated by averaging subtomograms (4 times binned) from a single long nucleocapsid without alignment. A cylindrical mask was generated in Dynamo according to the initial average, and used as alignment mask. Other masks were left as default. Subtomograms from the single long nucleocapsid were iteratively aligned against the initial reference in Dynamo, performing Euler angles and Cartesian shifts search. The resulting average of the single long nucleocapsid was used to align all subtomograms extracted from 20 tomograms, with the first two Euler angles restrained to not allow flipping during angular search. After the first round of rough alignment with a low-pass filter of ∼35 Å, the resulting average showed polarity. To determine the directionality of individual nucleocapsid, averages of subtomograms belonging to individual nucleocapsid with refined positions and angles were visually inspected. Subtomograms of nucleocapsids with opposite directionality with respect to the initial long nucleocapsid reference were flipped by modifying their Euler angles. After directionality check, rounds of iterative alignment were done until convergence. The refined coordinates of subtomograms were used for reconstructing 2 times binned subtomograms in Warp.

The subtomograms were then split into odd and even sets at 2 times binning for alignment using TOM/AV3 toolboxes. The odd and even sets were aligned independently using the average of each set as initial reference. Low-pass filter and angular search range/sampling parameters were adjusted based on the Fourier Shell correlation (FSC) plots after each round of alignment until convergence. After that, distance-based cleanup of subtomograms was done with MATLAB scripts to remove particles that were too close due to shift during alignment and the ones with higher constrained cross correlation (CCC) values were kept. The refined coordinates of subtomograms were used for reconstructing unbinned subtomograms (box size of 256^3^) with per-particle CTF models in Warp.

Unbinned subtomograms were averaged with refined Euler angles from TOM/AV3 alignment and intensity-inverted to generate initial reference for RELION 3D auto-refinement. Then, standard 3D auto-refinement was performed without symmetry, with a soft-edged sphere-multiplied cylindrical mask which was generated using relion_helix_toolbox (outer diameter 220 Å, sphere percentage 0.55). A 30 Å lowpass-filtered map generated from TOM/AV3 alignment was used as an initial reference. 1,209 subtomograms led to a 10 Å map with no symmetry applied. Helical parameters were estimated with relion_helix_toolbox, resulting in an average helical rise of 4.46 Å and helical twist of -27.08° (left-handed, ∼13.3 subunits per turn). Next, helical symmetry was imposed in RELION 3D auto-refinement with local symmetry search, resulting in the refined average helical rise of 4.16 Å and helical twist of -27.16°. After RELION classification with helical symmetry imposed, two classes of distinct helical parameters were separated: the majority class (77.7%, 4.21Å/-27.17°, map at 6.5 Å after auto-refinement) and the minority class (21.3%, 3.51Å/-26.9°, map at 7.3 Å after auto-refinement).

Positions and orientations of RELION symmetry-expanded subtomograms of the majority class were next used for refining tilt series alignment and CTF models in M software.^23^^,^^24^ After resolution convergence was reached in M (4.8 Å), new non-symmetry expanded subtomograms were reconstructed in Warp. One more round of RELION 3D auto-refinement imposing helical symmetry (which remained the same after M refinement) resulted in a final map of 4.5 Å for the majority class within the reconstruction mask. The minority class was refined in a newer version of M which allowed to set helical symmetry parameters. After resolution convergence was reached in M, subtomograms were again reconstructed in Warp. One more round of RELION 3D auto-refinement imposing helical symmetry (remaining the same after M refinement) resulted in a final map of 6.3 Å for the minority class within the reconstruction mask with the 0.143 criterion.

Maps used for structural analysis were obtained by filtering to the estimated resolution using the RELION Post-processing module with the same sphere-multiplied cylindrical mask. Visualization of density maps were done with UCSF Chimera or ChimeraX. FSC plots were obtained with the RELION Post-processing module using the same sphere-multiplied cylindrical mask. Local resolution estimation was also done in RELION with the Local resolution module providing the B-factors obtained from post-processing jobs and the masks. Local resolution maps were rendered with Chimera.

#### Subtomogram averaging of straight nucleocapsids *in situ*

For subtomogram averaging of the relatively straight nucleocapsids *in situ* (at ∼6 h of 30 μM As(III) and ∼6 h of 1 mM As(V) stress), the coordinates of the Amira traced filaments were resampled with a MATLAB script to obtain equidistant points along the filament at every 5.2 nm (4 pixels of 8 times binned data, 13.4808 Å/pixel) and with a torsion angle (30°) assigned in similar way as for the isolated nucleocapsids (Figure S7D). These equidistant points were used as center points for extraction of subtomograms (box size of 32^3^) with Dynamo crop function. Alignment of 8 times binned subtomograms was done with Dynamo. Alignment started with the longest nucleocapsid in one tomogram exhibiting the longest persistence length. As described above, initial average was generated and used as reference for alignment of subtomograms in this longest nucleocapsid. The resulting average was used for alignment of subtomograms from four long nucleocapsids in this tomogram. To determine the directionality of the other three nucleocapsids, averages of subtomograms belonging to individual nucleocapsid with refined positions and angles were visually inspected. Subtomograms of nucleocapsids with opposite directionality to the longest nucleocapsid were flipped by modifying their Euler angles. After directionality check, alignment was re-done with subtomograms from these four nucleocapsids. The resulting average was next used to align all subtomograms from six tomograms for one round of angular and translational search. Directionality check was done for individual nucleocapsid as described above. Only nucleocapsids showing clear directionality amongst those longer than 100 nm were kept for further processing. Subtomograms of nucleocapsids with opposite directionality to the longest nucleocapsid were flipped by modifying their Euler angles. Next, two more rounds of iterative alignment were done until convergence. Similarly, distance- and CCC-based cleaning of subtomograms was done with MATLAB scripts. The refined coordinates of subtomograms were used for reconstructing 4 times binned subtomograms in Warp.

The 4 times binned subtomograms were then aligned using TOM/AV3 toolboxes. After three rounds of iterative alignment, distance- and CCC-based cleanup of subtomograms was done. The refined coordinates of subtomograms were used for reconstructing two times binned subtomograms (box size of 128^3^) with per-particle CTF models in Warp.

The 2 times binned subtomograms were used for RELION 3D auto-refinement, following similar procedures as described for the isolated nucleocapsids. 2,178 subtomograms led to a 22 Å map with no symmetry applied. Helical parameters were estimated with relion_helix_toolbox and helical symmetry was imposed in RELION 3D auto-refinement with local symmetry search, resulting in the refined average helical rise of 4.24 Å and helical twist of -27.19°, and improved map resolution to 8.9 Å. RELION classification with helical symmetry imposed resulted in classes with very similar helical parameters, and were thus combined for further refinement. Positions and orientations of RELION helical symmetry-expanded subtomograms were next used for refining tilt series alignment and CTF models in M software. After resolution convergence was reached in M at 2 times binning, new non-symmetry expanded subtomograms were reconstructed in Warp. One more round of RELION 3D auto-refinement imposing helical symmetry resulted in a map of improved resolution at 7.2 Å and of the same helical parameters as the majority class in subtomogram averaging of isolated nucleocapsids (rise of 4.21Å and twist of -27.17°).

Unbinned subtomograms (box size of 256^3^) were then reconstructed in Warp at refined positions and subjected to RELION 3D auto-refinement. This step of 3D auto-refinement did not improve the resolution and classification resulted in very minor difference in helical parameters. The map was further refined in M which allowed to set helical symmetry parameters. After resolution convergence was reached in M, subtomograms were again reconstructed in Warp. One more round of RELION 3D auto-refinement imposing helical symmetry (remaining the same after M refinement) resulted in a final map of 6.5 Å within the reconstruction mask with the 0.143 criterion.

The map used for structural analysis was obtained by filtering to the estimated resolution using the RELION Post-processing module with the sphere-multiplied cylindrical mask.

#### Subtomogram averaging of curved nucleocapsids *in situ*

For subtomogram averaging of the curved nucleocapsids *in situ* (at ∼1 h of 0.5 mM As(III) stress), the coordinates of the traced nucleocapsids (done in Amira) were over-sampled with a MATLAB script to obtain uniformly distributed positions on the surfaces of nucleocapsid at every 2.7 nm (4 pixels of 2 times binned data), with initial Euler angles assigned at each surface position, and the new Z axis assigned perpendicular to the nucleocapsid axis (Figure S7A). These surface positions were used as extraction points for subtomogram reconstruction in Warp.

Alignment of 2 times binned subtomograms (box size of 36^3^) was done with TOM/AV3 toolboxes. The initial average was generated by aligning and averaging subtomograms of the longest nucleocapsid in a tomogram acquired with VPP, which has better contrast (signal-to-noise ratio) at low spatial frequency range. The VPP-average was used for alignment of subtomograms of all nucleocapsids in the two tomograms acquired without VPP. After 5 iterations of initial alignment, nucleocapsid directionality was checked by visual inspection of subtomogram averages of individual nucleocapsid. However, determination of the directionality was difficult due to poor signal-to-noise ratio of individual average. Thus, sampling was re-done at equidistant points along the central line of nucleocapsids and subtomograms were extracted for generation of averages of individual nucleocapsid, only for determination of directionality. Nucleocapsids showing clear directionality amongst those longer than ∼130 nm were kept for further processing. Subtomograms (oversampled) of nucleocapsids with opposite directionality to the VPP-average were flipped by modifying their Euler angles. Next, the VPP-average was again used to align the remaining 27,464 subtomograms (oversampled) until convergence. After that, distance- and CCC-based cleaning of clashing subtomograms was done with MATLAB scripts, with 4 pixels (2.7 nm) as a threshold. The refined coordinates of the 10,702 remaining subtomograms were used for reconstruction of subtomograms at 2 times binning in Warp with the same box size.

The re-extracted 2 times binned subtomograms were used for RELION 3D Classification using the average converted from TOM/AV3 alignment as a reference (filtered to 40 Å). Four classes were generated and one class (2882 particles) was discarded due to poor signal-to-noise ratio. The other three classes (2452, 2726, 2642 particles each) were separately refined, all resulting in maps of ∼30 Å in resolution. Further refinement using smaller masks did not improve maps, but resulted in too little content to be compared with higher resolution maps of isolated nucleocapsids. The maps used for structural analysis were obtained by filtering to the estimated resolution using the RELION Post-processing module with the refinement mask.

#### Model building

To build an atomic model for the majority class of the isolated nucleocapsids resolved to 4.5 Å, an initial model of MuV-N was generated with the homolog N protein of PIV5 (PDB: 4XJN) as a template using I-TASSER.^86^ The initial model was first fitted into the map of the majority class as a rigid-body, together with six RNA bases from the PIV5 model using Chimera. Only the core part of N (amino acid 3-405) was used for model building because the density for the C-terminal flexible region was missing. Then, the combined model of N and RNA was subjected to one round of automated refinement using Phenix^88^ (v 1.18-3845) with minimization_global, local_grid_search, simulated_annealing, and ADP strategies, and restraining rotamers, Ramachandran and secondary structures. Next, three copies of the refined N and RNA were generated and rigid-body docked in the cryo-EM density corresponding to the subunit at N_i+1_ position with respect to the original one (N_i_). Two additional subunits were docked into the upper turn in order to take interaction interfaces into account. After that, the tetramer model was per-residue manually refined in *Coot*^87^ (v0.9) using Peptide and Ramachandran restraints, with attention on the first monomer and molecular interfaces. Regions poorly fitted were deleted and re-built. N- and C-termini were checked and more residues added in case densities were seen. The RNA bases were modeled as poly-Uracils considering the averaging effect. Density modification strategy in Phenix.ResolveCryoEM was used to generate a map for aiding model inspection in *Coot*, but automated refinement and validation were done against the original map. The refined model of the first monomer was again copied and rigid body docked into the density map for a second round of automated refinement with Refine NCS operators on. Additional rounds of manual inspection and automated refinement were performed until convergence and Refine NCS operators was not used in the last round. The real-space cross-correlation of each residue to the map was calculated and plotted in order to evaluate the quality of the model (Figure S6C). The masked FSC between the original map and a simulated map calculated from the tetramer model at 0.5 cutoff gave a resolution estimate of 4.5 Å, similar to the estimate from the half map FSC 0.143 cutoff. MolProbity statistics were computed to ensure proper stereochemistry. Monomer of the model was used for rigid-body fitting into the map of straight nucleocapsids *in situ* (6.5 Å) with Chimera.

### Quantification and statistical analysis

The details of the quantification and all statistical analyses are included in figure captions or the relevant sections of method details.

## Data Availability

• Structures for initial model building and analysis of interaction interfaces were obtained from the Protein Data Bank (PDB) with accession codes PDB: 4XJN (PIV5 nucleocapsid-RNA complex) and PDB: 6V85 (PIV5 L-P complex), and the Electron Microscopy Data Bank (EMDB) under accession code EMD: 21095 (cryo-EM map of PIV5 L-P complex). Tomograms generated in this study are deposited in EMDB under accession codes EMD: 13165 (viral factory at non-stressed condition), EMD: 13166 (viral factory at 1 h of 0.5 mM As(III) stress), and EMD: 13167 (viral factory at 6 h of 30 μM As(III) stress). Cryo-EM maps are deposited under accession codes EMD: 13133 (majority class, isolated), EMD: 13136 (minority class, isolated), EMD: 13137 (straight cellular nucleocapsids). The associated model is deposited in the PDB under accession code PDB: 7OZR, and raw micrographs in the Electron Microscopy Public Image Archive under accession code EMPIAR: 10751 (isolated nucleocapsids). Mass spectrometry data are deposited in PRIDE. Data are available via ProteomeXchange with identifier PRIDE: PXD026799. Proteomics data for Figures 4 and S3 and sequencing data related to Figure S2A are deposited on Mendeley at https://doi.org/10.17632/4wzny9kwmn.1. All deposited data are publicly available as of the date of publication. • This paper does not report original code. • Any additional information required to reanalyze the data reported in this paper is available from the lead contact upon request.
