# Supplementary material for: Molecular mechanisms of stress-induced reactivation in mumps virus condensates
Source: Cell. 2023 Apr 27;186(9):1877–1894.e27. doi: 10.1016/j.cell.2023.03.015 (PMC10156176; doi:10.1016/j.cell.2023.03.015)
Supplement: Document S1. Tables S1–S3 [file mmc1.pdf]

Cell, Volume *186*

Supplemental information

Molecular mechanisms of stress-induced reactivation in mumps virus condensates

Xiaojie Zhang, Sindhuja Sridharan, Ievgeniia Zagoriy, Christina Eugster Oegema, Cyan Ching, Tim Pflaesterer, Herman K.H. Fung, Isabelle Becher, Ina Poser, Christoph W. Müller, Anthony A. Hyman, Mikhail M. Savitski, and Julia Mahamid

Table S1. Immuno-RNA FISH and qPCR probes/primers, related to STAR Methods

Probe/Primer Name	Sequences (5'->3')	Probe/Primer Name	Sequences (5'->3')
**FISH targeting L**		**FISH targeting N**	
L3972_1	TTGGAGCCAGATGACTTGG	N_1	TGTCGTCTGTGCTCAAAGC
L3972_2	AGTGATGGTCTAGAACGGG	N_2	GAGCGGTTCACGATAGAAC
L3972_3	CCATGTGTGCTCCTAAGG	N_3	GAACTTCAAGACAGGGGTG
L3972_4	AACGGTTATAGGAGGAGCC	N_4	TTAACACACCCAATCCCAC
L3972_5	AATGGAATCTGGCCGCCTA	N_5	TCTCACAGGGTAGGTGCA
L3972_6	ACTCTTCATGGCAATGCCC	N_6	GAGCCTGGTACATATAGGC
L3972_7	AAGCCTCATTGAGATGCGG	N_7	ATTCCAAATGCACGCGCCA
L3972_8	AGCAAGACTGGATGGGAG	N_8	GCTTTGCTTGCAGATGACC
L3972_9	CTATCGTGACGTGAATCGG	N_9	GTTGAAGGACAGCCATGTG
L3972_10	CTAGAGGATGACCGATTCG	N_10	ACCTTCGAACTCCAGTTGG
L3972_11	CACCCCACCTGTTTGAATG	N_11	TGCACTAGGGACCAAATGG
L3972_12	TCTCTATGTCGGTGATCCC	N_12	CGGTGAACTCACTAAGCTC
L3972_13	CAATCCTCCGTCAGATCC	N_13	GGGGGCTACCCATTGATA
L3972_14	TATAGTTTCCCCTCGTGGC	N_14	GTTATGCTATGGGAGTCGG
L3972_15	CCGAGAAGAAAGAGCAAGC	N_15	TCCAGATTGGGGTTGAGAC
L3972_16	AGCTACCACTGTAATGCGC	N_16	GGCCTAACTCCTGAGCAAA
L3972_17	GTTAATCCCATGCTCCGTG	N_17	CAACAACCTGCTGCCGTA
L3972_18	GAGGAACTCGCACAGTATC	N_18	GGAGTACAAGTACCAGCTG
L3972_19	GATCGCGAAGTAGTCATGC	N_19	AGACAGGGAGGTCAAACTG
L3972_20	CAGGTATTCACTGGAGGTG	N_20	AGAGCACAGCCTTTGCAAG
L3972_21	CCACTGTCAGCAAAGAAGC	N_21	GTCAGCAGTCAAAGTCTTCG
L3972_22	GTCCCATCGAGGGATTAGA	N_22	CTGCAGTCAAAATGCTAGG G
L3972_23	ATCGGGTCAGATGAGTGTG	N_23	GCAGATAGATCACCTGAAG C
L3972_24	TCACCTGGTTTTTCCTCCC	N_24	GAACGCTGTGAGATTGATG G
L3972_25	ATCAGCACTCAGGTTAGCG	N_25	CTGCCAATGAAATTGCTGCC
L3972_26	ACAGAGGAATCATGGCAGG	N_26	GGAACTCCTTACGTACATGC
L3972_27	GCATGGCCAGTTACCAAATG	N_27	GCCCAGAGGTTGATTCAAAC
L3972_28	GAAGAATCCCAGGTTCATGC	N_28	CTGCCTTCTTTCTCACTCTC
L3972_29	GCAAGAATTCCTAGTCTCCG	N_29	CGAGATATCGGAGAACAAG C
L3972_30	GCCATACTCATTTGGCCAAG	N_30	CAGTCCTAGATGCCCAAATG
L3972_31	GTATGCAATTGCCCACAGTG	N_31	CACTTATGCACGACCTTTCC
L3972_32	CTGTGAAGACCTGGCTTATG	N_32	CATCAGATCAATCGCATCGG
L3972_33	CCATTGTGGATACTAGGTCC	N_33	CGAACACGGAAACACTTTCC
L3972_34	GTTTCTTCTCTGTGCACAGC	N_34	AGGGTTCAATTCCGCCGGA

L3972_35	GAGATTCTCGACCTACTGAC	N_35	CGACCAACCTGCTGGATC
L3972_36	CATTATGAGACTTTGGGGCC	N_36	CAGCAAGGTCGCCTTGAAG
L3972_37	GGAGGAGCCTGATTAAACAG	N_37	AGATACATGCTGCAGCCGG
L3972_38	GAGCTTGAGTATGTCACCAG	N_38	AGACGGCAGGGGTTGCTA
L3972_39	GAGATAAAGGCTACAGGTCG	N_39	CACGAAGGCAACAAGGCAC
L3972_40	GATGGGTTTGAGATAGCAGC	N_40	AGGCTTGCAAGGGGAAGAG
L3972_41	GCTTCCTATCAACTGACCTC		
L3972_42	GGAGGTACCAAGTCATCATC		
L3972_43	CCAACTTGACCTTGATACCG		
L3972_44	GTTCACAAGCATCATGCTCC		
L3972_45	GTGAAAATGCAGCTGAGGAG		
L_end_1	TCCTCCAATCAACTTCTCC		
L_end_2	CTTGTGTGGTAAATCCGCC		
L_end_3	CCAGGAATGGCCATAACAG		
L_end_4	AACCAAGCAGGTGTTGACC		
L_end_5	CGAGACTCTGAAGAAGGAC		
L_end_6	CGCCAGCCTTGAGAAATAC		
L_end_7	AGTGGAGCCTCTATGTCAC		
L_end_8	TAGAGACTTTCTTGCCCGG		
L_end_9	CCTGGCTCAATGAACAGCA		
L_end_10	GGAGAGAGCTCAAGTACAC		
L_end_11	GCCACATTGGCAGTTGATC		
L_end_12	CCACATCCTCCTTTACAAC		
L_end_13	ATGGTTGGATCTGCCTGAC		
L_end_14	GAAATCCTAAAGGGCCAGG		
L_end_15	TCTTGCCTTCCCAAACTCG		
L_end_16	CCCTTACTAGCTCACCTTAC		
L_end_17	CTCCACACATAGCTTCACTG		
L_end_18	GTCACCTGAGGACAAATGTC		
L_end_19	CTAGGTTTGTCATCCACCTC		
L_end_20	CCCATCTATACTTGGCAGAG		
L_end_21	CCATATGGTACAACAGCCTG		
L_end_22	CAATAGTGGTGAGAATCCCC		
L_end_23	CTCCGATCCGAATAATCACG		
L_end_24	CCCACCTGAATTAGTGAGTG		
L_end_25	CTGGAGGAAGCGTGTTAAAC		
L_end_26	CTCTGAATAGGGCACGCAC		
L_end_27	GGGAGGTACTCCAAGCACA		
L_end_28	TGCTCACCCCGGATGTTGA		
L_end_29	GAGATCGATGATCAGGTAACG		

L_end_30	CTTGCTGTATAAGACCTGTCG
L_end_31	GACCCTGAACCACTTAGTTTG
L_end_32	GGTGGTTTAGATCAAATCCCG
L_end_33	GATGCTGTAGTTCAAGCAGAC
L_end_34	GTGTATATGACCTTGAGGAGG
L_end_35	CTCATCCACAATTAGTCACCC
**For cDNA generation**	
3'-UTR genomic forward	AGCTTGATCCTCACCTTCACC
L-gene antigenomic reverse	CATTTGGTAACTGGCCATGC
RNase P_reverse	GAGCGGCTGTCTCCACAAGT
**qPCR primers/probes**	
N-gene forward	GTATGACAGCGTACGACCAACCT
N-gene reverse	GCGACCTTGCT CTGGTATT
N-probe-LC610	LC610_CTGGATCTGCTGATCGACGAT_BHQ1
P-gene forward	GAGAAACCTGGAACCTCAAC
P-gene reverse	TTGGACCCAGCTGAGGCTC
P-probe-Cy5	Cy5_CTGCTCAAGGCCAGACAATCCAAGAGGA_BHQ2
F-gene forward	TCTCATCTATAGCAGGGAGTTATAT
F-gene reverse	GTTAGACTTCGACAGTTTGCAACAA
F-probe-FAM	6-FAM_AGGCGATTTGTAGCACTGGATGGAACA_BHQ1
RNaseP-gene forward	AGATTTGGACCTGCGAGCG
RNaseP-gene reverse	GAGCGGCTGTCTCCACAAGT
RNaseP-probe-HEX	HEX_TTCTGACCTGAAGGCTCTGCGCG_BHQ1

Table S2. Comparison of helical parameters of the MuV nucleocapsids obtained in this study to previously reported cryo-EM/ET paramyxoviruses nucleocapsids, related to Figure 6

Viruses	Nucleocapsids	Pitch / Rise (Å)	Twist (°)	Resolution (Å)	EMDB / PDB
MuV	Authentic, isolated	56 / 4.2	-27.2	4.5	This study 13133 / 7OZR
	Authentic, isolated	47 / 3.5	-26.9	6.3	This study 13136
	Authentic, in-cell	56 / 4.2	-27.2	6.5	This study 13137
	Recombinant [1]	53 / 4.0	-27.1	3.9	31368 / 7EXA
	Recombinant [1]	46 / 3.4	-26.8	3.6	31369 / 7EXA
	Virion associated [2]	67 / 5.3	-28.3	18.1	2630 / N.A.
MeV	Recombinant [3]	49 / 4.0	-29.2	4.3	2867 / 4UFT
	Recombinant [4]	49 / 3.9	-29.2	3.3	0141 / 6H5Q
	Virion associated [5]	64 / N.A.	N.A.	N.A.	1973 / N.A.
SeV	Recombinant [6]	54 / 4.1	-27.5	4.1	30066 / 6M7D
	Recombinant [6]	56 / 4.3	-27.4	4.6	30065 / 6M7D
	Recombinant [6]	53 / 4.1	-27.6	2.9	30129 / 6M7D

* 7EXA not released

Table S3. Cryo-ET data collection, refinement and validation statistics, related to STAR Methods

	Majority isolated (EMD-13133) (PDB 7OZR)	Minority isolated (EMD-13136)	Straight *in situ* (EMD-13137)	Curved *in situ*
**Data collection and processing**				
Magnification	81,000	81,000	53,000	42,000
Voltage (kV)	300	300	300	300
Detector	Gatan K2	Gatan K2	Gatan K3	Gatan K2
Electron exposure ($e^-/Å^2$)	102.5	102.5	158.6	145.0
Defocus range (μm)	2.5-3.5	2.5-3.5	1.75-3.25	3.25-3.5
Pixel size (Å)	1.6938	1.6938	1.631	3.3702
Symmetry imposed	Helical	Helical	Helical	C1
Final no. of segments/particles	939	270	2,178	7,820
Helical rise (Å)	4.21	3.51	4.21	NA
Helical twist (°)	-27.17	-26.9	-27.17	NA
Map resolution at 0.143 FSC (Å)	4.5	6.3	6.5	30
**Refinement**				
Initial model used (PDB code)	4XJN			
Model resolution at 0.5 FSC (Å)	4.5			
Map sharpening *B* factor ($Å^2$)	-56.3			
Model composition				
Non-hydrogen atoms	3320			
Protein residues	403			
Nucleotide residues	6			
B factors ($Å^2$)				
Protein	86.50			
Nucleotide	84.56			
R.m.s. deviations				
Bond lengths (Å)	0.005			
Bond angles (°)	0.709			
Validation				
MolProbity score	2.17			
Clashscore	16.12			
Poor rotamers (%)	0.00			
Ramachandran plot				
Favored (%)	92.77			
Allowed (%)	7.23			
Disallowed (%)	0.00			

Supplemental references

S1. Shan, H., Su, X., Li, T., Qin, Y., Zhang, N., Yang, L., Ma, L., Bai, Y., Qi, L., Liu, Y., et al. (2021). Structural plasticity of mumps virus nucleocapsids with cryo-EM structures. Commun. Biol. *4*, 833. https://doi.org/10.1038/s42003-021-02362-0.

S2. Cox, R., Pickar, A., Qiu, S., Tsao, J., Rodenburg, C., Dokland, T., Elson, A., He, B., and Luo, M. (2014). Structural studies on the authentic mumps virus nucleocapsid showing uncoiling by the phosphoprotein. Proc. Natl. Acad. Sci. U.S.A. *111*, 15208-15213. https://doi.org/10.1073/pnas.1413268111.

S3. Gutsche, I., Desfosses, A., Effantin, G., Ling, W.L., Haupt, M., Ruigrok, R.W., Sachse, C., and Schoehn, G. (2015). Structural virology. Near-atomic cryo-EM structure of the helical measles virus nucleocapsid. Science *348*, 704-707. https://doi.org/10.1126/science.aaa5137.

S4. Desfosses, A., Milles, S., Jensen, M.R., Guseva, S., Colletier, J.P., Maurin, D., Schoehn, G., Gutsche, I., Ruigrok, R.W.H., and Blackledge, M. (2019). Assembly and cryo-EM structures of RNA-specific measles virus nucleocapsids provide mechanistic insight into paramyxoviral replication. Proc. Natl. Acad. Sci. U.S.A. *116*, 4256-4264. https://doi.org/10.1073/pnas.1816417116.

S5. Liljeroos, L., Huiskonen, J.T., Ora, A., Susi, P., and Butcher, S.J. (2011). Electron cryotomography of measles virus reveals how matrix protein coats the ribonucleocapsid within intact virions. Proc. Natl. Acad. Sci. U.S.A. *108*, 18085-18090. https://doi.org/10.1073/pnas.1105770108.

S6. Zhang, N., Shan, H., Liu, M., Li, T., Luo, R., Yang, L., Qi, L., Chu, X., Su, X., Wang, R., et al. (2021). Structure and assembly of double-headed Sendai virus nucleocapsids. Commun. Biol. *4*, 494. https://doi.org/10.1038/s42003-021-02027-y.
